# The RNA secondary structure of androgen receptor-FL and V7 transcripts reveals novel regulatory regions

**DOI:** 10.1093/nar/gkae220

**Published:** 2024-03-30

**Authors:** Warren B Rouse, Van S Tompkins, Collin A O’Leary, Walter N Moss

**Affiliations:** Roy J. Carver Department of Biochemistry, Biophysics and Molecular Biology, Iowa State University, Ames, IA 50011, USA; Roy J. Carver Department of Biochemistry, Biophysics and Molecular Biology, Iowa State University, Ames, IA 50011, USA; Roy J. Carver Department of Biochemistry, Biophysics and Molecular Biology, Iowa State University, Ames, IA 50011, USA; Current Address: Departments of Biology and Chemistry, Cornell College, Mount Vernon, IA 52314, USA; Roy J. Carver Department of Biochemistry, Biophysics and Molecular Biology, Iowa State University, Ames, IA 50011, USA

## Abstract

The androgen receptor (AR) is a ligand-dependent nuclear transcription factor belonging to the steroid hormone nuclear receptor family. Due to its roles in regulating cell proliferation and differentiation, AR is tightly regulated to maintain proper levels of itself and the many genes it controls. AR dysregulation is a driver of many human diseases including prostate cancer. Though this dysregulation often occurs at the RNA level, there are many unknowns surrounding post-transcriptional regulation of AR mRNA, particularly the role that RNA secondary structure plays. Thus, a comprehensive analysis of AR transcript secondary structure is needed. We address this through the computational and experimental analyses of two key isoforms, full length (AR-FL) and truncated (AR-V7). Here, a combination of in-cell RNA secondary structure probing experiments (targeted DMS-MaPseq) and computational predictions were used to characterize the static structural landscape and conformational dynamics of both isoforms. Additionally, in-cell assays were used to identify functionally relevant structures in the 5′ and 3′ UTRs of AR-FL. A notable example is a conserved stem loop structure in the 5′UTR of AR-FL that can bind to Poly(RC) Binding Protein 2 (PCBP2). Taken together, our results reveal novel features that regulate AR expression.

## Graphical Abstract

**Graphical Abstract:**
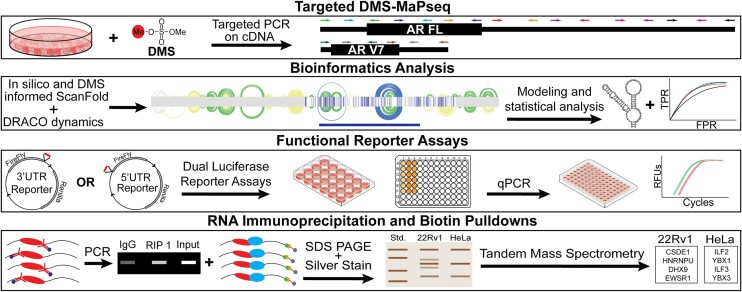

## Introduction

The androgen receptor (AR) is a ligand-dependent nuclear transcription factor that is a member of the steroid hormone nuclear receptor family (1). It is responsible for mediating the actions of androgens such as testosterone and dihydrotestosterone in various tissues and organs where it plays many roles in hormonal response during development and throughout life—including cell proliferation and differentiation (2–5). Regulation by AR primarily occurs through a direct interaction with androgen response elements (AREs) in the promoters of androgen regulated genes (6–8), however, AR can also regulate genes indirectly (9).

When AR becomes dysregulated it is the driving force of many human diseases including androgen insensitivity syndrome, Kennedy's Disease, and various cancers (10). Of particular interest is AR’s involvement in prostate cancer, which is the second leading cause of cancer related death among men in the United States (11). Generally, prostate cancer begins as androgen dependent, responding to changes in androgen levels, and can be treated through androgen-deprivation therapy (ADT) (12). Although ADT remains the primary therapy to prolong the life of patients (13), as prostate cancer progresses it often becomes androgen independent, or castration resistant, which currently has no effective treatment options (14). In these cases, cancer cells adapt to low androgen concentration through clonal selection of cells exhibiting AR amplifications or mutations, intracrine synthesis of testosterone from dehydroepiandrosterone (DHEA), ligand independent activation of AR, or bypassing of normal pathways to facilitate cancer progression (14–16). Interestingly, even in androgen independent cancer, AR expression is maintained or increased, and a direct correlation between AR mRNA levels and disease progression has been noted (17–20). Because of its importance in cancer, AR has been well studied at the protein level, but there is still much to be learned about its regulation at the mRNA level.

Post-transcriptional regulation of mRNA is often controlled by RNA secondary structure, which is known to affect splicing, translation, degradation, and localization (21). Contributions to post-transcriptional gene regulation primarily arise from structures found in the 5′ and 3′ untranslated regions (UTRs) (22,23). These roles include regulating translation initiation, stabilizing the transcript, acting as recognition sites for RNA binding proteins (RBPs), binding of microRNAs, and allowing or preventing access to single stranded RNA (24,25). An additional layer of regulation that is beginning to be explored is the ability of RNA to sample multiple conformations to fine tune interactions and respond to environmental cues and cellular conditions (21,26,27). A number of computational and experimental techniques have been devised to identify RNA structure and dynamics, determine interaction partners, and elucidate the components that modulate mRNA expression. Successful implementation of these techniques is and will continue to be crucial to uncovering functional mechanisms and unlocking a new world of possibilities in treating diseases that are currently ‘undruggable’ at the protein level (28,29).

**Figure 1.**
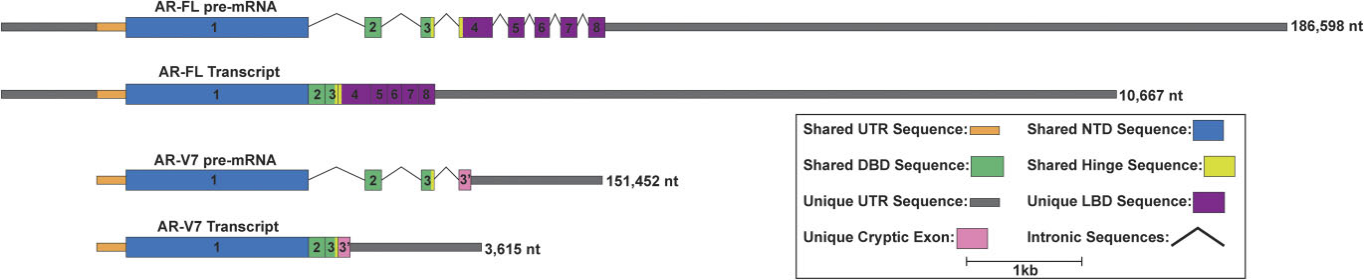
AR-FL and V7 pre-mRNA transcript diagrams drawn to scale. These diagrams show the differences in size and sequence/functional domains between the two isoforms. AR-FL pre-mRNA and mature transcript (ENST00000374690.9) are displayed on top and AR-V7 pre-mRNA and mature transcript (ENST00000504326.5) are displayed on the bottom. The AR-FL pre-mRNA is 186598 nt long, and the mature transcript is 10667 nt long, consisting of a 1126 nt 5′UTR, a 2763 nt CDS, and a 6778 nt 3′UTR. The AR-V7 pre-mRNA is 151452 nt long and the mature transcript is 3615 nt long, consisting of a 327 nt 5′UTR, 1935 nt CDS, and a 1353 nt 3′UTR. Although much shorter, the V7 isoform encodes a constitutively active version of the AR protein caused by a truncated ligand binding domain. Here, shared UTR sequences are displayed in orange and shared CDS sequences are displayed in blue, green, yellow, and purple. Unique UTR sequences are displayed in gray and unique CDS sequences are displayed in pink.

Several studies have identified RBPs, such as HuR (30–32), EBP1 (33,34), MSI2 (35), DDX3 (36) and HNRNPK (37) that interact with the UTRs of AR and elicit post-transcriptional regulation (38). Notably, Yeap et al (30) discovered a binding site for Poly(RC) binding protein 2 (PCBP2) in the 3′UTR of AR that increases the stability and translation of the mRNA. Others have identified and validated microRNA interaction sites throughout the MANE (matched annotation from NCBI and EMBL-EBI) isoform's extremely long 3′UTR (39–43). Undoubtedly, more is required for a full understanding of AR regulation. To this end, we identified intramolecular regulatory RNA structures of AR transcripts by experimentally and computationally mapping the static and dynamic RNA secondary structural landscapes of both the longest isoform (AR-FL) and the alternatively spliced and truncated isoform (AR-V7) (Figure 1). Select structures from AR-FL were tested for their potential roles in post-transcriptional gene regulation using in-cell assays and mutagenesis. Biochemical assays validated a novel interaction between PCBP2 and a conserved structure in the AR-FL 5′UTR, and identified several other RBPs in both UTRs that may play roles in post-transcriptional regulation.

## Materials and methods

### ScanFold

ScanFold is an RNA sequence scanning pipeline that deduces local structural stability, propensity for unusual sequence-ordered stability, and likely functional secondary structures by generating consensus structures where base pairs are weighted by their contribution to ordered structural stability. In brief, ScanFold is composed of a scanning step, ScanFold-Scan and folding step, ScanFold-Fold. Using ScanFold-Scan, a 120 nt scanning window is used to analyze the entire mRNA sequence of interest (here the AR-FL and V7 Ensembl transcripts were used as input). The sequence of each window is folded via RNAfold (44) to calculate its native minimum free energy (MFE) and associated base paired secondary structure. The native sequence in each window is shuffled 100 times, using mononucleotide shuffling, and folded to calculate an average randomized MFE value. The native and average randomized MFE values is then used to calculate the thermodynamic *z*-score. ScanFold-Fold analyzes all z-scores to generate a consensus secondary structure model based on paired nucleotides that recur across low *z*-score analysis windows (45). These structures are biased towards sequence-ordered stability, which suggests likely (evolved) functionality. Structures with low *z*-scores are extracted for further analysis. Metrics obtained from ScanFold include MFE, Δ*Gz*-score, and ensemble diversity (ED) (45,46). In this analysis, the following parameters were used: a 120 nt window size, a 1 nt step size, 100 randomizations per window, mononucleotide shuffling, 37°C temperature, competition of 1 (to demand that only one unique base pair per nucleotide is possible), and extraction of structures with *z*-score ≤ –1. All structure numbers, associated isoform and ensemble ID, transcript coordinates, sequence, in silico ScanFold structures, RNAfold refolded ScanFold structures, structure/sequence length, AU content, GC content, location within the transcript, and associated mass spec identified RNA binding protein can be found in Supplementary File S1. All ScanFold data can be found on our lab's structural database, RNAStructuromeDB (47,48). For more information on ScanFold see the original publications (45,46).

### Cell culture

Three different human cell lines were used in this study: HeLa (AR+), 22Rv1 (AR+), and DU145 (AR–). Expression of AR in all cells lines was validated by RT-qPCR (IDT, Hs.PT.56a.14520219). HeLa cells (ISU Hybridoma Facility) were maintained in DMEM, 22Rv1 cells (ATCC) were maintained in RPMI-1640, and DU145 (ATCC) cells were maintained in EMEM (ATCC) at 37°C in 5% CO_2_. All media was supplemented with 10% FBS, 100 U/ug per ml penicillin/streptomycin, and 2 mM l-glutamine. Unless otherwise specified, all media and supplements were obtained from Gibco/ThermoFisher. All cell lines were passaged at 80–90% confluence, used between passage 5–25, and regularly tested for mycoplasma (49).

### Dimethyl sulfate (DMS) probing of 22Rv1 cells

22Rv1 cells were grown to ∼80–90% confluence in 10 cm dishes. Cells were probed using a modified DMS-MaPseq protocol (50,51). A 2% (v/v) DMS solution was freshly prepared before treatment of each dish using 25% ethanol and 75% Dulbecco's phosphate buffered saline (DPBS). Growth media was removed from the cells before treating with 3 ml of DMS solution for 1 min at room temperature. The DMS was removed before cells were neutralized twice with 4.5 ml of Dithiothreitol (DTT) in DPBS (5 times molar excess to DMS). Cells were harvested with 1 ml of TRIzol (Invitrogen). DMS probing of cells was completed in triplicate.

### Isolation and purification of DMS probed RNA

RNA was isolated from each TRIzol sample using a minimally modified Direct-zol Plus RNA mini-prep kit protocol (Zymo). Briefly, TRIzol (Invitrogen) was loaded onto pre-spun QuantBio Heavy PLG tubes, 200 μl of 1-bromo-3-chloropropane (Sigma-Aldrich) was added to the sample and shook vigorously. After a 2 min room temperature incubation, the tubes were centrifuged at 12000×*g* for 15 min at 4°C. The aqueous phase was transferred to new 1.5 ml microcentrifuge tubes and an equal volume (∼500 μl) of 100% ethanol was added. Samples were loaded on Direct-zol Plus RNA mini-prep columns, and all downstream DNase treatment and washes were done according to manufacturer's protocol. After elution, a NanoDrop One (Thermo-Fisher) spectrum was taken to obtain the concentration of total RNA.

### PolyA selection and TGIRT-III reverse transcription

Purified RNA was poly(A) selected using the Poly(A) Purist MAG kit (ThermoFisher). A total of 70–80 ug of total RNA was used as input for each of the three DMS samples. After poly(A) selection, the average RNA concentration of the three samples was ∼1 μg or ∼1.3% of the input. This RNA was then used for reverse transcription (RT) with the TGIRT-III enzyme (InGex). Following a previously established protocol (51), 25 μl reactions were run using 550 ng of RNA and a 1:10 ratio of poly dT to random hexamer primers. RT products were diluted to 10 ng/μl for PCR amplification.

### PCR amplification and quality control

Using the cDNA generated from TGIRT-III RT, targeted amplification of the AR-FL and V7 isoforms was performed. PCR was completed using 20 μl Q5 (NEB) reactions with and without betaine (Sigma). For the FL transcript, 12 different tiled primer sets were used to amplify the 10667 nt transcript (Supplementary File S2). Amplicons ranged in size from 529 nt to 1198 nt with at least 50 nt overlap, and resulted in amplification of 9125 of 10667 nt, with the majority of unamplified sequence falling the in the 5′ and 3′UTRs. The unamplified region in the 5′UTR and the CDS had a GC content above 80%, whereas the region in the 3′UTR contained areas with GC content below 40%. For the V7 transcript, four different tiled primer sets were used to amplify the 3812 nt transcript (Supplementary File S2). Amplicons ranged in size from 627 nt to 1198 nt with at least 50 nt overlap, and resulted in amplification of 3521 of 3812 nt, with unamplified sequence falling in the CDS and at the end of the 3′UTR. The unamplified region in the CDS contained a region with GC content above 80%, whereas the region at the end of the 3′UTR was AT rich. All PCR products sizes were validated via agarose gel (Supplementary File S3) before determining their concentration on a Qubit® 2.0 Fluorometer. Using these concentrations, 20 ng of each product, per replicate, were pooled together and the combined concentration was determined using a Qubit® 2.0 Fluorometer.

### Library preparation and quality control

All six pooled PCR samples (3 FL and 3 V7) were then subjected to Illumina DNA library preparation following the manufacturers protocol. Here, input DNA concentrations ranged from 150 to 470 ng. Briefly, DNA was tagged with adaptor sequences and fragmented using Illumina's tagmentation process. Tagmented products were cleaned up and the resulting DNA was amplified with Illumina Nextera DNA UD Indexes (IDT) following Illumina's recommended PCR parameters. Amplified libraries were then cleaned following the standard DNA input protocol. Resulting library concentrations were determined using the Qubit® 2.0 Fluorometer and library sizes were determined using an Agilent 2100 Bioanalyzer. All libraries were determined to have a size between 500–600 nt (Supplementary File S3).

### Sequencing

All sequencing was completed on an iSeq100 benchtop sequencer (Illumina) using paired-end reads (150 × 150 nt). All libraries were first diluted to 1 nM with Illumina Resuspension Buffer (RSB) before combining the V7 and FL libraries together at equal volumes. Based on manufacturer's suggestion, the pooled libraries were further diluted to 75 pM with RSB before loading 20 μl into the sequencing cartridge and starting the sequencing run.

### Analysis of sequencing data with RNA Framework and DRACO

All 150 × 150 nt paired-end sequencing reads from the iSeq 100 were output as fastq files. The fastq files were uploaded to the Iowa State University High Performance Computing cluster and analyzed using the RNA Framework package (52), an all-in-one bioinformatics software package for analysis of next generation sequencing data generated through various types of RNA structure probing experiments. Fastq files first underwent quality control via fastqc to determine phred (quality) scores and adaptor content. All adaptor sequences were trimmed using the ‘cutadapt’ function before reanalyzing with fastqc. After completion of fastq file checks, Bowtie2 (53) was used to generate an index from the FL (ENST00000374690.9) and V7 (ENST00000504326.5) fasta files for read mapping. The ‘rf-map’ function was used to map all paired end reads to the Ensembl transcripts. All mapped bam files from the same library (FL or V7) were merged into a single bam file for both individual and combined processing. To obtain coverage and read depth information, samtools was used to analyze the bam files. The frequency of nucleotide mutations was counted using the ‘rf-count’ function and a rc file was generated. Mutations for either A and C or all nucleotides were normalized using the ‘rf-norm’ function and an xml file of reactivities was generated. The xml files were folded with ‘rf-fold’ and the -md flag to generate reactivity informed 120 nt and 600 nt max base pair span minimum free energy models (output as ct files). Using rf-fold with the -sh, -dp and -md flags, reactivity informed 120 nt and 600 nt max base pair span Shannon entropy and base pair probability files were generated. To analyze potentially dynamic regions, DRACO (54) was used. Using the mm file generated in the rf-count step, a json file was created for further processing. Using the json file and previous rc files, the ‘rf-json2rc’ function was used to generate a DRACO rc file. The DRACO rc file was then used as input for ‘rf-norm’ to generate individual xml files for each unique reactivity profile generated. All DRACO xml files were folded with ‘rf-fold’ to produce individual ct files. All xml files from RNA Framework and DRACO were converted to react files (folding constraints) using the python script ‘xml_reactivity_full_extract_batch.py’. The react files were then converted to heat maps for visualization of reactivities on structure models in Varna (55). For more information on the capabilities of RNA Framework visit (https://rnaframework-docs.readthedocs.io/en/latest/) and for DRACO visit (https://github.com/dincarnato/draco).

### Analysis of sequencing data with Shapemapper2 and SuperFold

All 150 × 150 nt paired-end sequencing reads from the iSeq 100 were output as fastq files. The fastq files were uploaded to the Iowa State University High Performance Computing cluster and analyzed using Shapemapper2 and SuperFold (56), a bioinformatic software pipeline for processing mutational profiling (MaP) data from RNA structure probing experiments and modeling the subsequent structures. Using the cat function, all forward and reverse read fastq files for each library were merged ($ cat FL_Fwd1 FL_Fwd_2 FL_Fwd_3 > out.fastq). Once merged, Shapemapper2 (56) was run with default parameters using the following command: $ shapemapper –name ‘outfilename’ –target fasta.fa –out ‘outfoldername’ –per-read-histograms –modified –R1 Read1_merged.fastq –R2 R2_merged.fastq. A log file was generated that reported the percent of nucleotides with a minimum read depth of 5000 (80% required to pass) and the number of highly reactive nucleotides (8% of nucleotides with depths above 5000 to pass). A map file, shape file, profile file, and several pdfs for data visualization were also generated. SuperFold (57) was then used to generate both a 120 nt and 600 nt max base pair span minimum free energy model from Shapemapper2 reactivity data. To run SuperFold, a slurm script was generated using the following commands ($ python Superfold.py maxPairingDist 600 ‘Shapemapper.map’ and $ python Superfold.py –maxPairingDist 120 ‘Shapemapper.map’). This resulted in generation of a log file, base pair probability file, Shannon entropy file, and multiple data visualization PDFs. For more information on the capabilities of Shapemapper2 visit (https://github.com/Weeks-UNC/shapemapper2), and for SuperFold visit (https://github.com/Weeks-UNC/Superfold).

### Incorporation of reactivity values into ScanFold

The reactivity profile and transcript sequence of both FL and V7 isoforms were analyzed with ScanFold. Reactivity files were first generated by conversion of xml files from RNA Framework using an in-house python script ‘xml_reactivity_full_extract_batch.py’. Reactivity files discussed above were used as pseudo-energy constraints to inform ScanFold predictions. These pseudo-energy constraints act as rewards or penalties during folding, favoring structures where nucleotides match their observed reactivity (unreactive or reactive). The following ScanFold command was used ($ python /path/to/ScanFold.py < Input.fasta> –out name < Input> –react < Input.react> –name < Input> –global refold). Here, the ‘–out name’ specifies an output directory name, ‘–react’ specifies the input react file name, ‘–name’ specifies the output file header names, and ‘–global refold’ enables global refolding of transcripts using DMS-informed ≤ –1 and ≤ –2 *z*-score DBN files as constraints.

### Comparison of DMS-informed and *in silico* structure models

DMS-informed ScanFold models, *in silico* ScanFold models, and DMS-informed MFE models for AR-FL and V7 mRNA sequences were compared to determine the effect of incorporating probing constraints on ScanFold models. This was done by calculating the PPV and sensitivity for each model through comparison of all predicted, ≤–1 *z*-score and ≤–2 *z*-score base pairs present in ScanFold CT files. This process utilized the script, ct_sensitivity_ppv.py, and all analysis steps and equations have been previously described (58). In addition to PPV/Sensitivity comparisons, a per nucleotide comparison of structures was completed for the different merged dataset models as well as the RNA Framework independent replicates (Supplementary Table S1). In addition to comparing the resulting structural models, the effect on the ScanFold per nucleotide z-score values (found in the ScanFold per nucleotide Zavg WIG file) were assessed between DMS-informed ScanFold (merged and replicate) and *in silico* ScanFold models. Here, a Pearson correlation assessment was conducted on a per method and per replicate basis comparing the DMS-informed ScanFold and *in silico* ScanFold per nucleotide *z*-score values (Supplementary File S4).

### Receiver operator characteristic analysis

All *in silico* ScanFold, DMS-informed ScanFold, and DMS-informed MFE models were compared to the different DMS reactivity profiles generated via RNA Framework and Shapemapper2 using a receiver operator characteristic (ROC) analysis script ‘roc.py’. The same ROC analysis strategy we previously reported (59) was used. In addition, a 100 nt sliding window ROC analysis was performed on all *in silico* ScanFold and DMS-informed ScanFold models. Briefly, the different z-score cutoff CT files for each secondary structure generation method were cross referenced to the different DMS reactivity profiles generated in this study. Reactivity data thresholds were sequentially set from lowest to highest at 1% intervals (i.e. 0–100%) where any associated nucleotide below the threshold was defined as being paired. Each reactivity threshold was then referenced to the CT files and a true positive rate (TPR) and false positive rate (FPR) were calculated. The TPR and FPR were plotted for each reactivity threshold to generate an ROC curve. Models that fit or agree with the reactivity profile will have a larger area under the curve (AUC), whereas models that are more ‘random’ compared to the reactivity data will have a smaller AUC.

### Covariation analysis

All FL and V7 structures with *z*-scores ≤–1 (89 and 25 structures respectively) were analyzed for covariation using the cm-builder Perl script (60). This script builds off the RNA Framework toolkit (52) and utilizes Infernal (release 1.1.2 (61)) to build and find covariance models for ScanFold predicted structures. The Infernal databases were created using a BLAST (62) search of the AR transcripts ENST00000374690.9 and ENST00000504326.5. Here, the NCBI Refseq database was searched using the following parameters: blastn, gap open 5, gap extend 2, reward 1, penalty –1, max target sequences of 5000. All pseudogenes and ‘like’ sequences were deselected and the resulting list was downloaded and used in the analysis. The resulting structural alignment files (in Stockholm format) were tested for covarying base pairs and analyzed with the CaCoFold algorithm and R-scape (version 1.5.16). Statistical significance was evaluated by the APC corrected G-test (63,64) using the default *E* value of 0.05. The power files generated were analyzed using an in-house script that bins according to the power of covarying base pairs into 0–0.1, 0.1–0.25 and >0.25. All input files, Stockholm alignments, R-scape/CaCoFold results, and power analysis data can be found in Supplementary File S5.

### Modeling of RNA structures and visualization of data

To model DRACO structures, unique dynamic profiles (determined by PPV and sensitivity) were spliced into the surrounding static profile using the python script ‘draco_react_splicer.py’. These contextualized react files were then used as constraints in RNAfold (44), a minimum free energy based structure prediction algorithm, with a 120 nt and 600 nt max base pair span. Visualization of 2D models and reactivity data was done using VARNA (55), and the Integrative Genomics Viewer (IGV) (65) was used for visualizing data tracks. All bioinformatics algorithms and tools used in this study can be found in Supplementary Table S2.

### Construct design and cloning

All constructs were designed based on the predicted secondary structures. All constructs tested were found in the 5′ and 3′UTR and designed for cloning into a modified pmirGLO plasmid (Promega). For HiFi cloning, a minimum of 15 nucleotides complementary to the upstream and downstream sequence surrounding the XhoI restriction enzyme site of the vector were added to each end of the sequences. A total of 10 WT and 6 mutant 5′UTR constructs and 4 WT 3′UTR constructs were designed for AR-FL. All mutations were made based on previous work by Yeap et al. (30). These sequences were ordered as GBlocks or Ultramers (IDT) for cloning. All cloning was completed in modified pmirGLO (Promega) dual luciferase plasmids designated pmirGLOi (3′UTR) and pmirGLO5i (5′UTR) (66). Briefly, the plasmid was modified by introduction of the rabbit B-Globin intron II into the Firefly (FF) and Renilla (RL) luciferase genes. The pmirGLO5i plasmid was also rearranged to move the multiple cloning site (MCS) from the 3′ end of FF to the 5′ end of FF. Cloning was done using the HiFi Assembly kit (NEB), with insertion of gBlocks or Ultramer oligonucleotides (IDT) into XhoI digested plasmids. HiFi reactions were run at 50°C for 30 min, followed by transformation in NEB-5*α* competent *Escherichia coli*. Colony PCR was used to validate proper insertion, and sequences were validated by Sanger Sequencing (Iowa State DNA Facility). A full list of constructs, the cloning strategy, and primers used can be found in Supplementary File S6.

### Cell plating, transfection, and harvesting

HeLa cells were trypsinized from a 10 cm dish at 80–90% confluence. Cells were counted using a hemocytometer and plated at 25000 cells/well in a 96-well dish (six biological replicates per construct) for dual luciferase assays and at 150000 cells/well in a 24-well dish (3 biological replicates per construct) for qPCR. Cells were transfected 24 hours later with experimental or control (empty pmirGLOi or 5i) plasmids using Lipofectamine 3000 (Invitrogen). Respectively, 5 and 25 ng of dual luciferase plasmid was transfected into each well of the 96- and 24-well plates with 95 or 475 ng of pUC19 plasmid filler, respectively. Cells were supplemented with fresh DMEM 24 hours post-transfection and analyzed (dual luciferase assay) or harvested (for RNA) 24 hours after supplementation.

DU145 and 22Rv1 cells were trypsinized from T75 flasks at 80–90% confluence prior to nucleofections using modified protocols from Lonza Biosciences and Spisák *et al.* (67), respectively. Briefly, cells were counted using a hemocytometer, and the volume needed for 400000 cells per transfection was centrifuged at 200×*g* for 3 min. The DU145 and 22Rv1 cell pellets were resuspended in supplemented SE and SF nucleofection reagent (Lonza Biosciences), respectively, and 20 μl was aliquoted to individual tubes. To each tube, 1 μl of the appropriate plasmid (400 ng) was added, and each 21 μl sample was transferred to an individual well of a nucleocuvette. A Lonza 4D nucleofector X-unit was run with protocols CA-137 for DU145 and EN-120 for 22Rv1. Cells were incubated at room temperature for 10 min before resuspending them in 479 μl of pre-warmed media (per well) and plating 400 μl in a single well of a 24-well dish and 100 μl into a single well of a 96-well dish (three biological replicates per construct). Cells were supplemented with fresh media 24 hours after nucleofection and processed (as with HeLa cells) 24 hours after supplementation.

### Dual luciferase assays

A Promega Dual Luciferase kit was used following the manufacturer's protocol. In brief, cells in the 96-well dish were washed with DPBS (Gibco) before lysing with 1x passive lysis buffer (PLB). Lysate from each well was transferred to an opaque white 96-well dish for recording luminescence using a dual injecting GloMax Explorer (Promega). The Relative Response Ratio (RRR) was calculated by dividing the light units from Firefly luciferase by those of Renilla luciferase on a per-well basis. The RRR was normalized to the mean of the vector control and the resulting values were plotted as the mean ± standard deviation. Using a two tailed, equal variance *t*-test, the significance of changes caused by wild type (WT) constructs were compared to the vector control, and the significance of changes caused by mutant constructs were compared to the WT version. Significance *P*-values of <0.05 were used.

### RNA isolation and cDNA preparation

Cells were harvested from the 24-well dish using 400 μl of TRIzol (Invitrogen). RNA was isolated following the same modified Direct-Zol RNA mini-prep (Zymo) protocol used in the isolation of DMS probed RNA above. cDNA was generated following the Superscript III (Invitrogen) RT manufacturer's protocol. Here, between 200 ng and 1 μg of RNA was used with random hexamer (IDT) priming. For quality control, no RT controls were completed for all samples. All cDNA samples were diluted with water to ∼5 ng/μl based on RNA input to normalize for quantitative PCR (qPCR) input.

### qPCR

Quantitative PCR was performed with 1 μl of the diluted cDNA, cPrimeTime® primer/probes (IDT) designed to overlap the introduced intron for each of the Firefly and Renilla luciferase genes (Firefly: forward 5′-ACAAAACCATCGCCCTGATC-3′, reverse 5′-ATCTGGTTGCCGAAGATGG-3′, probe 5′6-FAM/ACCGCTTGT/ZEN/GTCCGATTCAGTCAT/3′IABkFQ; Renilla: forward 5′-CCTACGAGCACCAAGACAAG-3′, reverse 5′-ACCATTTTCTCGCCCTCTTC-3′, probe 5′SUN/CACGTCCAC/ZEN/GACACTCTCAGCAT/3′IABkFQ), and PrimeTime® Gene Expression Master Mix on a QuantStudio3 (Thermo-Fisher). Ct values were calculated using the automatic threshold detection settings of the QuantStudio Design & Analysis desktop software (v1.5.1). The ddCt method was employed with Renilla and empty pmirGLOi as references to obtain the average fold expression (2^−ΔΔCT^) and standard deviation. Using a Student's *t*-test, the significance of changes caused by WT constructs were compared to vector control, and the significance of changes caused by mutant constructs were compared to the WT version. Significance *P*-values of <0.05 were used. Translational efficiencies were calculated by dividing the normalized RRR by the average fold expression (2^−ΔΔCT^) and propagating the error. Statistics included a two-tailed, equal variance *t*-tests with a significance *P*-value of <0.05.

### Preparation of whole-cell lysates

HeLa, DU145 and 22Rv1 cells were trypsinized, counted, and centrifuged for 3 min at 200×*g*. Cells were washed once with ice cold DPBS, pelleted again, and resuspended at 10^7^ c/ml in ice cold polysome extraction buffer (PEB: 20 mM Tris, pH 7.5; 100 mM KCl; 5 mM MgCl_2_; 0.5% NP-40) (68) containing 1 mM PMSF, HALT, 200 U/ml RNaseOUT (ThermoFisher). The resuspended pellet was incubated on ice for 15 min prior to Dounce homogenization (methanol-cleaned glass). The homogenates were spun at 15000×*g* for 10 min at 4°C prior to collection of the supernatant. Lysate concentrations were determined using a BCA assay (ThermoFisher).

### *In vitro* transcription and RNA 3′ end desthiobiotinylation

Plasmids containing AR-FL structure 32 (AR 32) as well as ultramers for AR-FL 4 WT and Mut 1–3 (4 WT and Mut 1–3) were used as PCR templates to make T7-promoter containing DNA Supplementary File S3. T7 recognition sequences were incorporated into forward strands using primers found in Supplementary File S2. PCR products were *in vitro* transcribed with T7 RNA polymerase (Invitrogen) to generate RNA. RNA was heated to 75°C for 15 minutes and cooled to room temperature before 100–500 ng was used to verify the correct RNA product size via urea-PAGE (National Diagnostics Urea-Gel SequaGel) and SYBR Green II RNA Gel Stain (ThermoFisher) (Supplementary File S3). *In vitro* transcribed RNA was 3′ end labeled using the RNA 3′ end desthiobiotinylation kit (Pierce) following the manufacturer's protocol. Briefly, 50 pmol of each RNA was added to individual 30 μl ligation reactions. The ligation reaction was incubated at 16°C overnight before adding 70 μl of water and 100 μl of chloroform:isoamyl alcohol (24:1). The mixture was vortexed briefly and centrifuged at 12 000×*g* for 5 min to separate. The aqueous phase was removed and transferred to a new tube before adding 10 μl of 5M NaCl, 1 μl of glycogen, 2 μl of glycoblue (ThermoFisher), and 300 μl of ice-cold 100% ethanol. This mixture was precipitated for 2 hours at –20°C before centrifuging at ≥13 000×*g* for 15 min at 4°C. The supernatant was removed, and pellets were washed with 300 μl of ice cold 70% ethanol. The pellets were air dried for 5–10 min before resuspension in 20 μl of UltraPure water (Invitrogen). Labeling efficiency was determined to be ∼75% using a dot blot with a pre-biotinylated RNA control (Supplementary File S3).

### Biotin RNA pulldowns

An initial biotin pulldown was performed using a Magnetic RNA Protein Pull Down kit (Pierce) following the manufacturer's protocol. Briefly, the 50 pmol of each 3′ end desthiobiotin labeled RNA was incubated with an equal amount of nucleic acid compatible streptavidin magnetic beads (50 pmol and 50 μl) in RNA Capture Buffer for 30 min at room temperature to form RNA-bead complexes. The RNA-bead complexes were then mixed with protein-RNA binding buffer, 50% glycerol and 188 μg of 22Rv1 cell lysate before rotation for 1 hour at 4°C. Beads were washed three times with 1X wash buffer (Pierce) to remove unbound proteins. Washed beads were submitted for on-bead digestion and LC–MS/MS analysis (Iowa State Protein Facility). MS results were searched against only human protein data in the UniProt database. Proteins pulled down in the unlabeled RNA bead control samples were removed from all other samples. The Mascot scores of remaining proteins were averaged and those with a score of three times the average were analyzed in more detail. A secondary biotin pulldown was performed on structure 32 following the same protocol. Here, structure 32 was incubated with 188 and 172 μg of 22Rv1 and HeLa cell lysate, respectively. After the final washes, the RNA and captured proteins were eluted from the beads using 50 μl of biotin elution buffer (Pierce). Beads were collected on the magnetic stands and the supernatant was added to a new tube with 2× Lameli BME buffer. Samples were run on a 4–15% SDS PAGE gel (Biorad) before being silver stained. Unique bands were excised from the gel and LC–MS/MS analysis was performed (Iowa State Protein Facility). MS results were searched against only human protein data in the UniProt database, and all resulting proteins were analyzed.

### SDS-PAGE and silver stain

All pulldown samples were thawed at room temp and spun down at 10000×*g* for 1 min. Loading dye was added to each sample and heated to 95°C for 5 min. Samples were spun down before 50 μl of sample and 5 μl of protein standard (BioRad) were loaded on a TGX 4–15% precast gel (BioRad) and run for 40 min at 200 V. While the gel was running all glassware was washed with methanol. The gel was removed from the cassette, trimmed, placed in a covered dish, and rocked with 400 ml of fixative solution (BioRad) for 30 min at room temperature. The fixative was removed, 400 ml of water was added, and the gel was rocked for 10 min at room temperature. The water was removed, and a second water incubation was performed for 10 min. All water was removed from the dish before the silver stain solution was added and rocked at room temperature for 11 min. The reaction was quenched by removing the staining solution and rocking in 400 ml of 5% acetic acid for 20 min. The annotated gel can be found in Supplementary File S3.

### RNA immunoprecipitation

All RNA immunoprecipitations were done following a modified protocol from Lee and Moss *et al.* (69). Briefly, eight 10 cm dishes each of both HeLa and 22Rv1 cells were rinsed twice with 10 ml of DPBS before lysis with 500 μl (per two 10 cm dishes) of cold RIPA lysis buffer (50 mM Tris–HCl; pH 8.0; 1 mM EDTA; 150 mM NaCl; 100 μM Na_3_VO_4_; 1.0% NP-40; 1.0% sodium deoxycholate; 0.1% SDS) containing protease/RNase inhibitors (1:100 HALT; 1 mM PMSF; 300 U/ml RNaseOUT). A cell lifter was used to transfer the lysing cells to 15 ml conical tubes on ice. Lysates were sonicated on ice four times at 50% amplitude for 5 sec prior to centrifugation to clear the lysate (15 000×*g*, 10 min, 4°C). The supernatant was diluted 2.5-fold with detergent-free lysis buffer containing 300 U/ml of RNaseOUT, followed by pre-clearing with 100 μl of protein A/G-PLUS agarose beads (Santa Cruz Biotechnology) for 1 hour at 4°C. For each sample, 1.4 ml of HeLa lysate and 1.6 ml of 22Rv1 lysate was incubated with either anti-hnRNP E2 (sc-101136, Santa Cruz Biotechnology), anti-HuR (sc-5261, Santa Cruz Biotechnology) or mouse IgG antibody (sc-2025, Santa Cruz Biotechnology) for 1 hour at 4°C on the rotisserie. A/G-PLUS agarose beads were then added (25 μl) for an additional hour at 4°C on the rotisserie. For input samples, 25% of the volume added for each antibody of the pre-cleared lysate was set aside for RNA isolation (350 μl of HeLa and 400 μl of 22Rv1). Beads were washed three times with cold DPBS containing RNaseOUT and PMSF (1 ml each) before TRIzol was added (to the input as well). Samples were then incubated for 15 min at room temperature prior to storage at –80°C or used directly in RNA isolation. Total RNA isolation was done using Zymo RNA Clean & Concentrate kit, per manufacturer's protocol. Input lysate was eluted at a higher volume to make the input 10% at equal volumes to the immunoprecipitation (IP) samples for reverse transcription (RT). No RT controls were done using the input lysate. Total RNA, random hexamers (IDT) and Superscript III (ThermoFisher) were used for RT. Semi-quantitative PCR was used to determine protein enrichment in the 3′UTR and the 5′UTR as the regions could not efficiently be amplified via qPCR. As a positive control, the primers from Yeap *et al.* (30) were tested on all RIP samples, and for the experimental region in the 5′UTR all IP samples except HuR were tested. PCR cycle numbers were optimized used 23, 25 and 30 cycles for each cell line used. Cycle numbers of 25 and 30 were found to be optimal for 22Rv1 and HeLa cells, respectively.

## Results

### Targeted DMS-MaPseq of AR mRNA in a human prostate cancer cell line model

**Figure 2.**
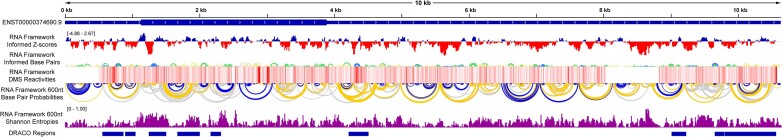
All displayed data tracks were generated from RNA Framework informed predictions of AR-FL and are presented as seen in the Integrative Genomics Viewer (IGV). The AR-FL transcript cartoon is displayed at the top for spatial orientation of all other data. Below the transcript cartoon is the RNA Framework informed ScanFold *z*-scores with positive values in blue and negative values in red. Below the *z*-scores, the first arc diagram represents the RNA Framework informed ScanFold predicted base pairs with *z*-score > 0, >–1, >–2 and ≤2 color in white/gray, yellow, green and blue respectively. Below the ScanFold arc diagram, the DMS reactivities are shown as a heat map on a scale of 0–1 where 0 is white, 1 is dark red, and intermediate values are shades of red. Below the DMS reactivity heat map, the second arc diagram represents the base pair probabilities calculated from an minimum free energy fold using RNA Framework reactivities and a 600 nt max base pair span constraint, with probabilities >80%, 30–80% and 10–30% displayed in blue, gold and gray respectively. Comparison of the ScanFold and base pair probability arc diagrams demonstrates the general agreement between low *z*-score structures and highly probable base pairings. Below the base pair probability arc diagram, the purple bar graph represents the Shannon entropies calculated using RNA Framework reactivities and a 600 nt max base pair span constraint. The blue bars at the bottom represent dynamic regions identified by DRACO.

**Figure 3.**
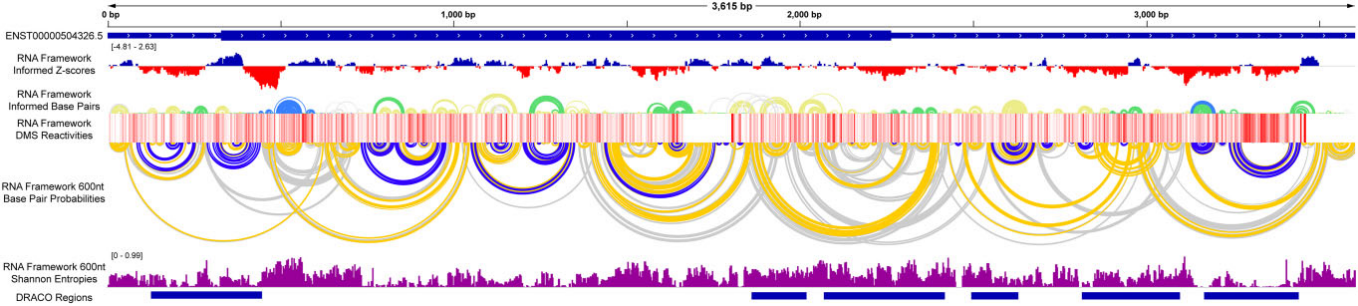
All displayed data tracks were generated from RNA Framework informed predictions of AR-V7 and are presented as seen in the Integrative Genomics Viewer (IGV). The AR-V7 transcript cartoon is displayed at the top for spatial orientation of all other data. Below the transcript cartoon is the RNA Framework informed ScanFold *z*-scores with positive values in blue and negative values in red. Below the *z*-scores, the first arc diagram represents the RNA Framework informed ScanFold predicted base pairs with *z*-score >0, >–1, >–2 and ≤2 color in white/gray, yellow, green and blue respectively. Below the ScanFold arc diagram, the DMS reactivities are shown as a heat map on a scale of 0–1 where 0 is white, 1 is dark red, and intermediate values are shades of red. Below the DMS reactivity heat map, the second arc diagram represents the base pair probabilities calculated from an minimum free energy fold using RNA Framework reactivities and a 600 nt max base pair span constraint, with probabilities >80%, 30–80% and 10–30% displayed in blue, gold and gray respectively. Comparison of the ScanFold and base pair probability arc diagrams demonstrates the general agreement between low *z*-score structures and highly probable base pairings. Below the base pair probability arc diagram, the purple bar graph represents the Shannon entropies calculated using RNA Framework reactivities and a 600 nt max base pair span constraint. The blue bars at the bottom represent dynamic regions identified by DRACO.

To gain experimental support for RNA secondary structure models, we employed targeted DMS-MaPseq (51) in 22Rv1 cells to analyze both the longest isoform, AR-FL (FL), as well as the prostate cancer-associated truncated isoform, AR-V7 (V7). Biological triplicates for each library were analyzed as merged and independent datasets using the RNA Framework and Shapemapper2/SuperFold pipelines. These tools were designed to process RNA structure probing data from mutational profiling (MaP) experiments and model the resulting structures. For the merged datasets, a total of ∼8.8 million and ∼3.4 million reads were obtained for FL and V7 libraries, respectively. All reads were mapped to the Ensembl (70) transcripts, ENST00000374690.9 (FL) and ENST00000504326.5 (V7) using bowtie2 within either RNA Framework or Shapemapper2. Using RNA Framework, FL and V7 isoforms were found to have mean read depths of 119317 and 130355 and read coverages of 87.8% and 95.7%, respectively. Reactivity profiles from RNA Framework were generated for FL and V7, and the average reactivities were 0.428 and 0.480, respectively (Supplementary Table S3). Each biological replicate for the FL and V7 isoforms were also processed by RNA Framework and found to have consistent metrics (Supplementary Table S4). Implementation of the orthogonal approach, Shapemapper2, on merged datasets yielded very similar average read coverages and reactivities (Supplementary Table S3). Due to the read requirements of Shapemapper2, biological replicates were not analyzed independently. For both analysis methods, normalized per nucleotide reactivities showed that A and C nucleotides were the predominant reactive nucleotides (Supplementary Figure S1), reflecting the specificity of DMS for these residues. Comparing the reactivities of merged datasets from both methods revealed a correlation coefficient (Pearson) of 0.840 and 0.907 for FL and V7, respectively (Supplementary File S7). Comparison of replicate reactivities to merged reactivities showed similar correlations; however, much lower correlations were seen between replicates. A 100 nt sliding window analysis of replicate reactivity data revealed a relationship between coverage and reactivity correlation; however, the disparity in reactivity correlation did not affect the predicted structures (Supplementary Figures S2 and S3; and Supplementary Table S1). Chemical reactivity data from both merged and replicate experiments (DMS profiles) were used as constraints to guide ScanFold predictions. These ‘pseudo-energy’ constraints act as rewards or penalties during folding, favoring structures where nucleotides match their observed reactivity (unreactive or reactive). Additionally, models incorporating reactivity information, along with base pair probabilities and Shannon entropies, were generated for the merged datasets using RNA Framework and SuperFold (Figures 2 and 3). Interestingly, base pairs with low *z*-scores (indicating ordered stability) matched those with high predicted base pair probabilities. Notably, several of the regions with the greatest predicted base-pairing inconstancies between methods and replicates were found in regions deemed to be dynamic using DRACO (Supplementary Figures S4 and S5; and Supplementary File S8).

To assess the effects of DMS probing constraints on global ScanFold metrics, purely *in silico* ScanFold was used to predict RNA secondary structures with sequence-ordered stability. Supplementary Table S5 shows the comparison between *in silico* ScanFold and merged RNA Framework informed metrics, demonstrating the highly consistent results between the two. The same analyses using Shapemapper2 informed predictions produced similar results (Supplementary Table S5). Correlation analyses of z-scores offered further evidence of this consistency (Supplementary File S4). Using the merged dataset, comparison of RNA Framework informed ScanFold base pairs, at different z-score cutoffs, to reactivities generated with RNA Framework showed that lower z-score pairs (comprised of nucleotides with the greatest propensity for ordered stability) have slightly lower and fewer reactivities (Supplementary Figure S6A). Thus, nucleotides exhibiting a more negative *z*-score are more likely to be inaccessible due to their presence in stable/ordered structure, whereas nucleotides with positive *z*-scores and the highest mean reactivity values are more likely ordered to be *un*structured. When the RNA Framework base-pair probabilities (generated from informed MFE predictions) are considered, lower *z*-scores correspond to higher base pair probabilities (Supplementary Figure S6B). This supports the predictive ability of ScanFold by highlighting how lower z-score structures are more likely to form based on their uniquely ordered and stable nature. The same analyses were done using both Shapemapper2 and SuperFold, revealing consistent trends for reactivities and base pair probabilities (Supplementary Figure S7). Collectively, these data show that RNA Framework and Shapemapper2 perform similarly.

### Experimental data supports ScanFold models

To assess the effects of DMS probing constraints on ScanFold model generation, DMS-informed and *in silico* ScanFold models of FL and V7 were analyzed using PPV and sensitivity metrics as well as per nucleotide pairing comparisons. In these analyses, DMS-informed models were used as the reference and *in silico* models were used as the predicted. PPV indicates the fraction of consistent base pairs between models by comparison to the total number of predicted pairs, allowing for assessment of how specific the predicted models are compared to the reference. Sensitivity indicates the fraction of consistent base pairs between models by comparison to the total number of reference pairs, assessing how correct the base pairing predictions are. Pairwise comparisons of *in silico* and informed ScanFold data, binned according to the reference z-score, were performed for both AR isoforms (Supplementary Table S6). For the FL model *in silico* and informed ScanFold PPV comparisons, all but two comparisons were above 0.7, indicating that the majority of *in silico* base pairs, at different z-score cutoffs, are the same as those predicted using probing constraints. The only comparisons with PPV values <0.7 were *in silico* vs RNA Framework and Shapemapper2 informed ScanFold models at a –2 *z*-score cutoff. When looking at sensitivity values from the same *in silico* and informed comparisons, the results match that of PPV, indicating that the majority of experimentally supported base pairs were predicted by *in silico* only models. We also see that *in silico* vs RNA Framework informed models produce higher PPV and sensitivity values than *in silico* vs Shapemapper2 informed models. Comparison of the informed models for AR-FL yielded the expected results, showing the highest and lowest PPV and sensitivity for the -1 and -2 z-score models, respectively (Supplementary Table S6). For V7 models, the PPV and sensitivity yielded less predictable results. *In silico* and informed ScanFold PPV and sensitivity comparisons revealed all but two comparisons were >0.6. In this case, the low PPV values were for *in silico* versus RNA Framework informed models at –2 and –1 *z*-score cutoffs, and the low sensitivity value was for the *in silico* versus RNA Framework informed model at a –2 *z*-score cutoff. In contrast to FL, we also see that *in silico* vs Shapemapper2 informed models produce higher PPV and sensitivity values than *in silico* vs RNA Framework informed models. Comparison of the informed models for AR-V7 yielded different results than AR-FL, showing the highest and lowest PPV and sensitivity for the no filter and –2 *z*-score models, respectively (Supplementary Table S6). Direct comparison of ScanFold structures generated using *in silico* only, RNA Framework, and Shapemapper2 methods revealed highly consistent results. The pairing consistency ranged from 77% to 99% across all *z*-score cutoffs (Supplementary Table S1). This agreement suggests that the observed differences in PPV and sensitivity for FL-V7 might be attributed to subtle, per-nucleotide *z*-score variations. These variations can lead to minor pairing changes in shorter transcripts, ultimately influencing the specific pairs considered at each *z*-score threshold.

We also performed static and sliding window ROC analyses to assess how well the different models agree with DMS reactivity data using RNA Framework and Shapemapper2 DMS reactivity profiles in concert with the –2, –1 and no filter *z*-score CT files for all structure models (see Materials and methods). Static ROC analysis of both AR isoforms using either *in silico* ScanFold models and RNA Framework reactivities or RNA Framework informed ScanFold models and RNA Framework reactivities produced very consistent AUC values for each *z*-score bin (Supplementary Figure S8). These AUC were between 0.6 and 0.8, indicating some agreement between ScanFold models and RNA Framework reactivities for FL and V7. To further investigate this, we used a 100 nt sliding window analysis to identify regions of the transcripts with high and low AUC values. Here, we found that regions of less stable, higher *z*-score structures were the main contributors to the low static AUC values (Supplementary Figures S9 and S10). Although this trend holds true across most of the transcript, some low *z*-score structures such as 6–7 in the 5′UTR of AR-FL are represented by high quality data that have lower than expected AUC values in the windowed analyses (Supplementary Figure S9B and Supplementary File S9). This is also the case for structure 14 in the CDS and structure 32 in the 3′UTR of AR-FL. Interestingly, these regions correlate with DRACO structural dynamics data providing evidence that the low AUC values are potentially the result of structural heterogeneity. Additional static and sliding window ROC analyses were completed for FL and V7 using combinations of all available models and reactivities, which produced variable AUC values and levels of support for the different models and reactivities that were compared (Supplementary Tables S7 and S8; Supplementary Figure S8; Supplementary File S9).

### Regions of conformational dynamics identified within AR mRNAs

In addition to determining the static structures of AR mRNAs, we also looked at their potential for conformational dynamics. To do this we used DRACO, an algorithm designed to deconvolute RNA structure heterogeneity from mutational profiling (MaP) data (54). With the capability of MaP experiments to introduce multiple mutations on a single cDNA, DRACO can extract multiple unique reactivity profiles from the same dataset allowing for identification of structural heterogeneity (potentially dynamic RNA secondary structures). Analysis of the FL and V7 RNA Framework reactivities by DRACO revealed 9 and 6 regions of potentially dynamic structures dispersed across the transcripts, respectively (Figures 2 and 3). For FL, 5 of the dynamic regions encompassed –2 *z*-score structures while the other 4 regions encompassed either –1 or weakly negative *z*-score structures. For V7, all 6 regions encompassed either –1 or weakly negative *z*-score structures. Here, the focus was on regions that contained –2 *z*-score structures of FL including one in the 5′UTR, one in the CDS, and two in the 3′UTR.

Within the 5′UTR, unique reactivity profiles were predominantly found for the region encompassing structures 6 and 7. Based on the PPV and sensitivity analysis of these profiles (Supplementary File S10), only profiles resulting in structural changes were folded using a 600 nt pairing constraint. This resulted in four clusters of conformations that maintained either part or all of the ScanFold predicted structures. With a 120 nt pairing constraint, all structures were nearly identical to each other and the predicted ScanFold hairpins (Supplementary Figure S11). Fewer reactivity profiles were found in the CDS for the region encompassing structures 13–15. Unique profiles from PPV/sensitivity analysis (Supplementary File S10) were analyzed for structural differences using a 600 nt pairing constraint. Here, 3 different multi-branch conformations containing either part or all of the ScanFold predicted structures were identified. The 120 nt pairing constraint maintained similar conformations as the 600 nt constraint, but as individual hairpins and a small multi-branch loop (Supplementary Figure S12). This is not surprising due to the high ensemble diversity of the structures and the nature of the CAG repeats that allows for base pair slipping. Unique reactivity profiles were also found in the 3′UTR for the region containing structures 31–34. Unlike any of the other dynamic regions, these profiles all formed distinct conformations regardless of the base pair constraints used. Although distinct conformations formed, either part or all of the ScanFold predicted structures were still maintained. Here, the predominant changes were seen in structure 32 where the predicted hairpin is restructured into a multi-branch loop (Supplementary Figure S13). The large terminal loop of structure 32 and the A-rich nature of the region could explain this potential conformational switch. Near the end of the 3′UTR additional reactivity profiles were found for the region containing structures 86–89. The unique profiles clustered into 3 different multi-branch loop conformations. Similar to all other regions, when a 600 nt base pair constraint was used the conformations maintained either part or all of the ScanFold predicted structures. Like the CDS, when a smaller base pair span was used, similar conformations were maintained, but as individual hairpins and small multi-branch loops rather than one large domain. Here, the predominant changes were seen downstream of the low z-score structures (Supplementary Figure S14). Although structures 6–7, 13–15 and 31–34 were maintained between reactivity profiles, differences in pairing and z-scores were seen between RNA Framework and Shapemapper2 informed ScanFold in these regions (Supplementary Figure S4 and Supplementary File S8), offering further evidence of structural heterogeneity.

### ScanFold identified structured regions regulate gene expression *in vitro*

**Figure 4.**
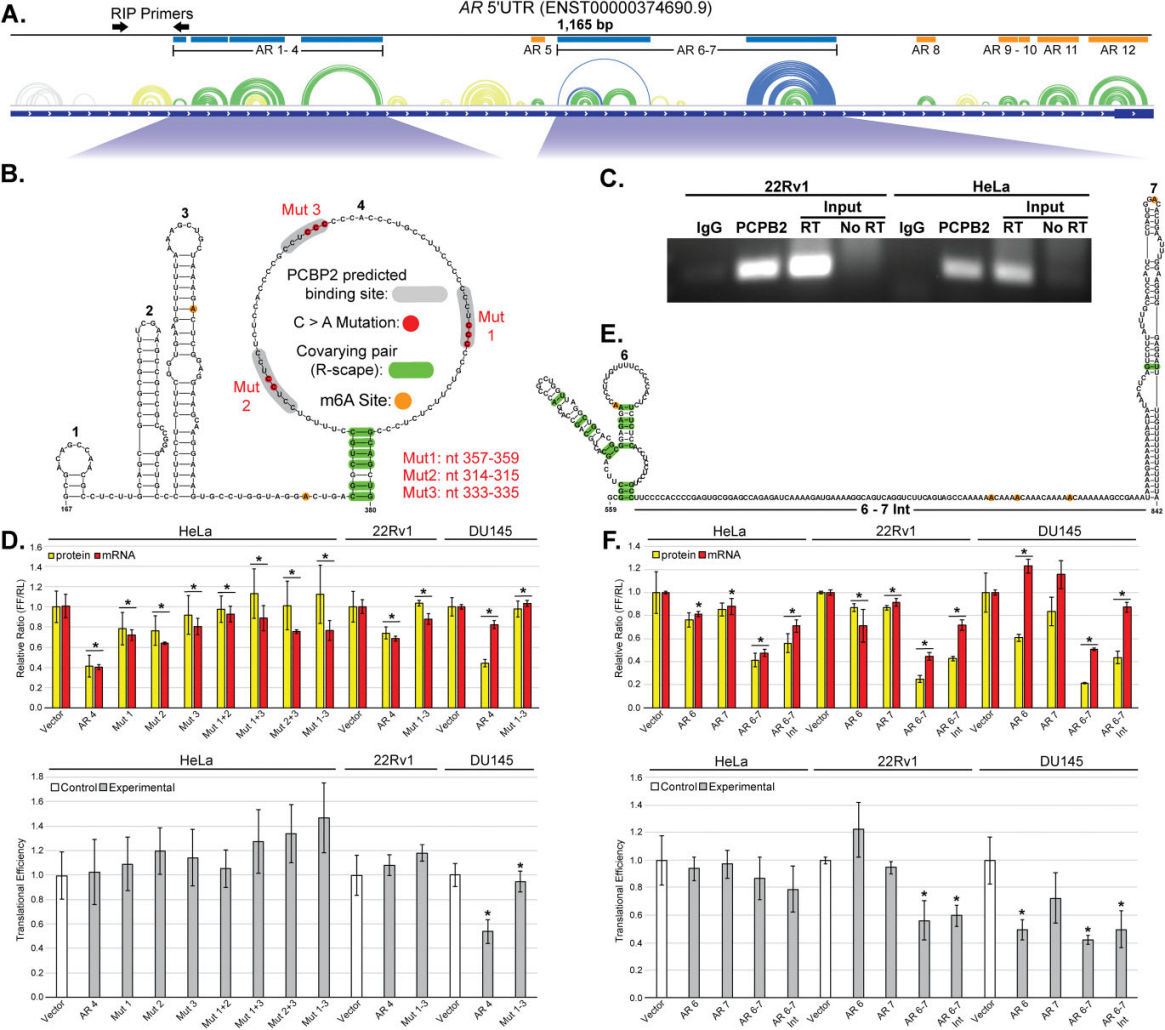
AR-FL 5′UTR functional assay results. Structure function data for AR-FL 5′UTR structures 1–4 and 6–7 in HeLa, 22Rv1, and DU145 cell lines. (**A**) The entire AR-FL 5′UTR is shown at the top with ScanFold predicted structures represented as an arc diagram above the gene cartoon. Low *z*-score structures (blue and green arcs) are annotated with their number and a blue or orange box. Structures annotated with blue boxes are expanded and represented below the arcs as 2D models. Black arrows represent the location of RIP primers. (**B**) The individual structures of AR 1–4 are numbered and annotated with all relevant data including two m6A modifications (orange circles), six covarying base pairs (green bars) and three predicted PCBP2 binding sites (gray highlight). Within the predicted PCBP2 binding sites, C > A mutations (red nucleotides) were made to ablate the potential interaction for functional testing. Mutant 1–3 were made at transcript positions 357–359, 314–315 and 333–335, respectively. (**C**) Results of semi-quantitative PCR on RIP samples from 22Rv1 and HeLa cells. Primers targeted a 141 nt region of the FL 5′UTR, near structure 4 (top diagram). On the left, the results from 22Rv1 cells shows an enrichment in PCBP2 protein binding compared to the IgG control at 25 cycles. On the right, the results from HeLa cells shows an enrichment in PCBP2 protein binding compared to the IgG control at 30 cycles. In both cases the intensity of the PCBP2 band was far greater than that of IgG and similar to that of the 5% Input (total lysate) sample. (**D**) Dual luciferase, and translational efficiency results for structures 1–4 in HeLa, 22Rv1, and DU145 cells. Changes in protein (yellow) and mRNA (red) levels compared to vector or WT control can be seen in the top bar graphs. Here, AR 4 was compared to vector and all mutants were compared to the WT AR 4. Changes in translational efficiency seen in the lower bar graphs following the same comparisons as protein and mRNA. Here, vector controls are represented in white and experimental constructs are in gray. Asterisks represent a *P*-value <0.05 determined using a two-tailed Student's *t*-test. (**E**) The individual components of structure 6–7 are numbered and annotated with all relevant data including five m6A modifications (orange circles) and 13 covarying base pairs (green bars). (**F**) Dual luciferase, and translational efficiency results for structures 6–7 in HeLa, 22Rv1 and DU145 cells. Changes in protein (yellow) and mRNA (red) levels compared to vector can be seen in the top bar graphs. Changes in translational efficiency compared to vector are seen in the lower bar graphs. Here, vector controls are represented in white and experimental constructs are in gray. Asterisks represent a *P*-value <0.05 determined using a two-tailed Student's *t*-test.

**Figure 5.**
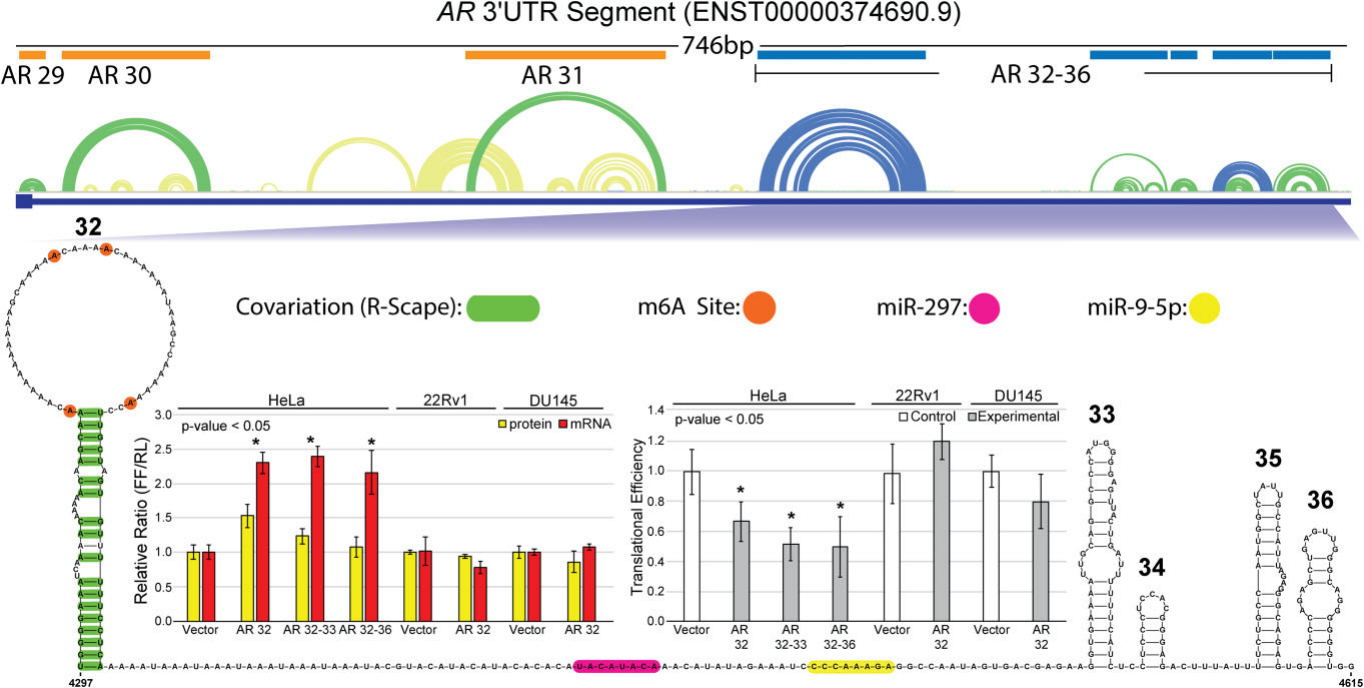
AR-FL 3′UTR functional assay results. Structure function data for AR-FL 3′UTR structures 32–36. A 746 nt fragment at the beginning of the AR-FL 3′UTR is shown with ScanFold predicted structures represented as an arc diagram above the gene cartoon. Low z-score structures (blue and green arcs) are annotated with their number and a blue or orange box. Structures annotated with blue boxes are expanded and represented below the arcs as 2D models. The individual hairpins of the 2D model are numbered and annotated with all relevant data including four m6A modifications (orange circles) and eighteen covarying base pairs (green bars) on structure 32, and a miR-297 site (pink) and a miR-9-5p site (yellow) for the single stranded region between structure 32 and 33. These structures were tested for function via dual luciferase assays and qPCR in HeLa, 22Rv1 and DU145 cells. The changes in protein (yellow) and mRNA (red) levels compared to vector control can be seen in the left bar graph. Using the protein and mRNA levels, translational efficiency was calculated and plotted in the right bar graph. Asterisks represent a *P*-value <0.05 determined using a two-tailed student T-test.

With the majority of low *z*-score structures remaining consistent between the different analysis methods, cm-builder (60) was used to identify structures with statistically significant covarying base pairs. These covarying base pairs indicate that co-mutation of paired nucleotides has occurred across species to preserver the structure. Of the 89 FL structures with *z*-score ≤–1, 25 showed evidence of at least one base pair with statistically significant covariation. Of these, there were 7 in the 5′UTR, 4 in the CDS and 14 in the 3′UTR. For V7, 9 of the 25 structures were similarly identified with 2 in the 5′UTR, 3 in the CDS, and 4 in the 3′UTR. All covariation data can be found annotated in Figures 4 and 5 and in Supplementary File S5.

Combining information from the results above, 10 structures from AR-FL (6 from the 5′UTR and 4 from the 3′UTR) were assessed via dual luciferase reporter assays for effects on RNA stability and/or translation in human cells. Three different human cell lines were used: HeLa, 22Rv1 and DU145. Beginning in HeLa with 5′UTR structures 1–4 (AR 1–4) (Figure 4A and B), a statistically significant and proportional (no change in translational efficiency [TE]) decrease in protein and mRNA were observed compared to vector control (Supplementary File S11). The same comparison with structure 4 (AR 4) removed (just structures 1–3 present) caused essentially no change in protein level but a statistically significant decrease in mRNA, resulting in an increased TE. Furthermore, AR 4 on its own reproduced the results from AR 1–4, indicating its sufficiency in reducing the level of mRNA. These data indicate that AR 4 was responsible for a reduced mRNA level and plays a role in regulating translational efficiency in concert with 1–3.

AR 4 has 6 covarying base pairs in a short stem capped with a large CU-rich loop (Figure 4B). The loop contains several consensus PCBP1/2 binding sites (30). Due to its high expression level in our cell lines (71), its upregulation in aggressive prostate cancers (72), and its ability to regulate other human UTRs (73,74), we focused our analyses on PCBP2. PCBP2 was confirmed to interact with AR mRNA by RNA immunoprecipitation (Figure 4C). In an attempt to disrupt putative protein-RNA interactions, mutations of these consensus sites were tested (similar to Yeap *et al.* (30)) both individually and in combinations (Figure 4D). All individual and combined mutations caused statistically significant increases in protein and mRNA compared to WT AR 4 but non-significant increases in TE. To determine whether this effect was cell-type specific, the WT AR 4 and triple mutant construct (Mut 1–3) were tested in 22Rv1 and DU145 cells. Again, the WT construct decreased both protein and mRNA compared to vector control in each, and the triple mutant increased both protein and mRNA compared to WT. Unlike in HeLa or 22Rv1 cells, AR 4 reduced translational efficiency in DU145.

We also wanted to examine a region identified as structurally dynamic in the 5′UTR of AR-FL, so two additional structures, AR 6 and 7 (Figure 4A and E), were tested both together and individually (Figure 4F). Both contain covarying base pairs and are separated by a stretch of unstructured, A-rich nucleotides that were previously shown to be methylated (m^6^A) (75). Like AR 1–4, when AR 6 and 7 were tested together (AR 6–7) a reduction was seen in both the level of protein and mRNA (compared to vector) in all three cell lines. Not only was this effect greater in the 22Rv1 and DU145 lines, but translational efficiency was also significantly reduced. Interestingly, when just the *un*structured intervening region (AR 6–7 Int) was tested, it alone was sufficient to reduce luciferase expression, mRNA, and translational efficiency (compared to vector), but true to all three cell lines, the level of mRNA was greater than when AR 6 and 7 were also present. These data suggest that this dynamic region regulates AR expression.

With an abundance of structures to choose from in the 3′UTR of AR-FL, we looked at two low z-score clusters composed of potentially dynamic structures AR 32–36 and AR 86–87 (Figure 5; Supplementary Figure S15). AR 32–33 and 32–36, which are near the CDS and contain several validated microRNA sites (39,40,43), were effective at reducing translational efficiency despite a doubling of mRNA in HeLa cells (Figure 5). The miRNA sites may have contributed to reduced translation, but structure 32 alone–with 18 covarying pairs, four sites of m^6^A modification, but no known miRNA binding sites–sufficiently elicited the same results. AR 32 had no effect compared to vector control in the 22Rv1 and DU145 lines, however, suggesting that other interacting factors play a role in mediating the cell-type specific effects. Closer to the end of the AR-FL 3′UTR, AR 86–87 was also tested in HeLa cells and revealed a significant increase in the mRNA level but no effect on protein levels (Supplementary Figure S15).

### Identification of putative novel AR protein interactions

**Figure 6.**
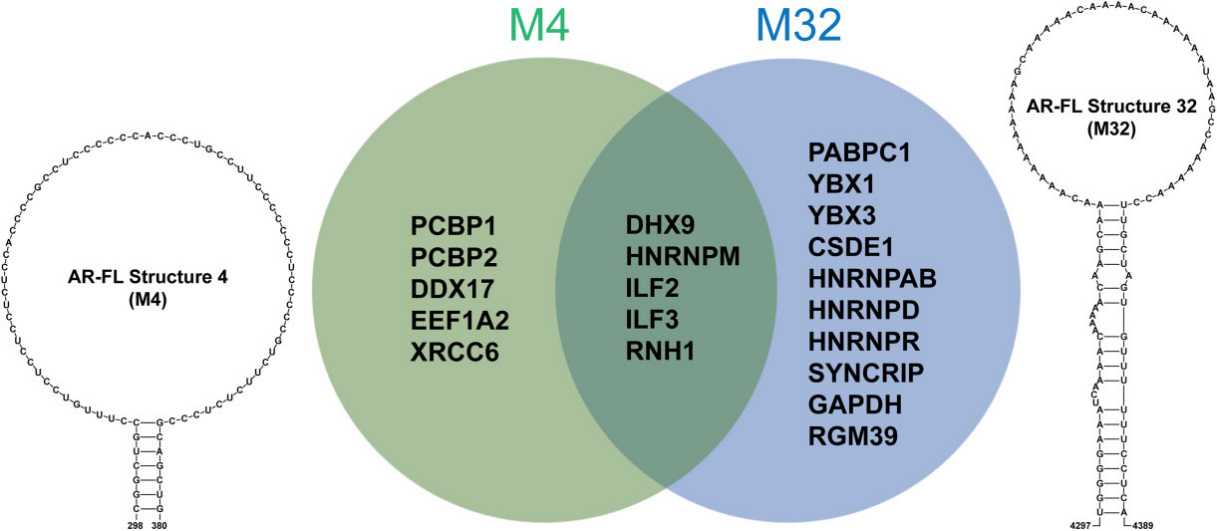
The strongest identified proteins (Mascot scores 3X > background for each sample) from biotin RNA pulldowns using synthetic AR-FL structure 4 WT and structure 32 in 22Rv1 cell lysates are compared using a Venn diagram. Unique proteins for structure 4 WT are within the light green circle, unique proteins for structure 32 are in the light blue circle, and common proteins identified in both are in the dark green intersecting region. The secondary structure models of structure 4 and structure 32 are on the left and right side of Venn diagram, respectively.

To identify protein binding factors involved in the regulation of AR mRNA, we performed RNA-pulldown experiments using *in vitro* transcribed and biotinylated RNA, 22Rv1 cellular lysate, and mass spectrometry (MS). Here, the 3′UTR region in AR-FL identified by Yeap *et al.* (30) to bind PCBP2 was used as a positive control, a 25mer poly(A) sequence was used as a negative control, and a non-labeled RNA was used as a pulldown control. For the identification of novel protein interactors, we used two functionally relevant and conserved structured RNAs as bait (both having long stems capped by large single stranded loops): AR 4 and AR 32, which correspond to ScanFold identified structures found in the 5′ and 3′UTRs, respectively (Figures 4 and 5). Mass spectrometry of proteins captured in the biotin pulldowns resulted in hundreds of putative hits in each sample (Supplementary File S12). To focus on only the most robust results, proteins identified in each sample were filtered based on their Mascot scores (a summary of quality control metrics, such as the percentage of matching peptides used to make an ID). Limiting our attention to proteins identified with very strong Mascot scores (>3× the ‘background’ average score of all hits) resulted in 10 putative interacting proteins for AR 4 and 15 for AR 32 (Figure 6). Of these, 5 proteins were common to both RNAs, 5 were unique to AR 4, and 10 were unique to AR 32. Notably, PCBP1 and PCBP2 were both found in the AR 4 pulldown samples, where the endogenous interaction of PCBP2 with AR 4 was confirmed via RIP (Figure 4C). The interaction with AR 4 is interesting, as we found this 5′UTR element to suppress AR expression (Figure 4B), whereas the PCBP2 interaction with the 3′UTR of AR was previously found to be stabilizing (30). STRING analysis of the 20 identified strong hits found a tight cluster of nodes with significantly more interactions than expected, implying biological connections (Supplementary File S12). Likely biological processes for these connected proteins were identified and found to be consistent with roles in post-transcriptional regulation of AR: e.g. regulation of mRNA translation, stabilization, processing, etc. Interestingly, biological roles in viral genome replication and viral/host translation through interactions with IRES stem-loop RNA structures (76,77) were identified for PCBP2 and CSDE1 (Cold Shock Domain Containing E1).

Additional pulldowns were conducted in multiple cell lysates to identify potentially unique RNA binding proteins involved in the differential changes caused by structure 32 in our functional assays. Here, we used *in vitro* transcribed and biotinylated AR 32 RNA, a non-labeled RNA pulldown control, and both 22Rv1 and HeLa cell lysates. The proteins captured in the pulldown were ran on an SDS-PAGE gel and silver stained to identify band patterns unique to each lysate (Supplementary File S3). Three prominent bands from the 22Rv1 lysate were excised and proteins were identified via mass spectrometry. Using the approximate molecular weights from the gel to assess reasonability of returned hits, one potential protein was identified from band 1 and two potential proteins were identified from band 3. Similar to what was seen in the previous pulldown experiment, the band 1 protein was found to be DExH-box helicase 9 (DHX9), and the band 3 proteins were found to be Cold Shock Domain Containing E1 (CSDE1) and Interleukin Enhancer Binding Factor 3 (ILF3) (Supplementary File S12).

### Isoform specific changes in RNA secondary structure

Through our analysis of AR-FL and V7 RNA secondary structure, we have identified many structures that are both common and unique between the two isoforms. Interestingly, all of the AR-FL structures that were tested and found to be functional are not present in the truncated AR-V7 isoform (Figure 1). Through the truncation of the 5′UTR, alternative splicing of the CDS, and replacement of the 3′UTR only 13 of the 89 low *z*-score structures of FL are present in V7. Of the structure shared between isoforms, 5 are from the 5′UTR, 8 are from the CDS, and none are from the 3′UTR. Comparing changes in structural propensity for each RNA, we found differences in average z-scores for FL and V7 of –0.58 and –0.35 respectively. These changes become more notable when partitioning results between the UTRs and coding sequences (CDS). Here, the average *z*-scores of the FL 5′UTR, 3′UTR and CDS are –0.66, –0.25 and –0.70 compared to –0.50, –0.13 and –0.62 for V7. Similarly, RNA structural dynamics differ between AR-FL and AR-V7, with less dynamic regions identified in V7. Interestingly, two different V7 dynamic regions were identified across the 5′UTR-CDS junction whereas none were identified for FL.

## Discussion

### *In silico* only ScanFold compares favorably with structure probing data

Using targeted DMS-MaPseq we determined the RNA secondary structure landscape of two low abundance transcripts, AR FL and V7, in 22Rv1 prostate cancer cells. This powerful technique gave excellent signal, sufficient for the determination of structural dynamics. Through the use of RNA Framework, DMS reactivity data was generated and used to inform ScanFold and RNAfold structure predictions. Use of the programs Shapemapper2/SuperFold also showed congruency with that of RNA Framework, however the RNA Framework data was more amenable to structural dynamics deconvolution using DRACO. PPV/sensitivity and ROC analysis of all structure predictions provided valuable insight into ScanFold's prediction and modeling capabilities with and without probing data. Several low z-score structures identified in our predictions were also shown to be functional via dual luciferase and qPCR. Through mass spectrometry and RNA immunoprecipitation, these structures were shown to interact with many RBPs that support our functional readouts.

We have rigorously analyzed all DMS sequencing data from biological replicates and merged datasets and found good correlation between the different methods as well as the replicates versus the merged datasets (Supplementary File S7). Although the comparison of per replicate reactivity data to each other yields low correlation, we have found that all replicates exhibit very similar DMS signal, read coverage, and read depth. Analysis of coverage data in concert with both per nucleotide reactivity standard deviation data and 100 nt sliding window Pearson correlation analysis of reactivities revealed a direct relationship between high coverage regions, low per nucleotide reactivity standard deviation, and high reactivity correlation (Supplementary Figures S2 and S3). Even in the low coverage areas, the least variable reactivities between replicates exist in more stable, lower z-score regions. While every site of reactivity may not match perfectly in magnitude, structure predictions yield results that are highly consistent—also reflected by the results using the merged data. A comparison of ScanFold output files generated from each replicate dataset were found to have between 85 and 99% consistency in pairings (Supplementary Table S1).

Using all reactivity data we demonstrated that *in silico* ScanFold compares favorably to DMS-informed ScanFold (58,59) by performing in-depth assessments of how probing data affects ScanFold results. Incorporation of any individual replicate or merged DMS reactivities as pseudo-energies into ScanFold predictions caused an increase in MFE compared to *in silico* predictions (Supplementary Table S5) however this is not surprising, as pseudo-energies penalize the overall predicted MFE proportionate to the reactivities (27,58). No other significant changes in structure were noted (Supplementary Table S1). PPV/sensitivity and ROC analysis allowed for assessment of *in silico* ScanFold's performance with and without probing data as well as how the probing data influenced model generation. Depending on the transcript isoform, the PPV and sensitivity results varied significantly (Supplementary Table S6); however, the per nucleotide consistency in predicted structure between all methods remained high (Supplementary Table S1). The differences in PPV and sensitivity can largely be explained based on changes in per nucleotide z-scores caused by inclusion of probing constraints. These z-score shifts, in turn, affected the pairs considered at different z-score cutoffs. In addition, many of the pairing discrepancies affecting PPV and sensitivity either fell in dynamic regions, regions of higher z-score, or repeat regions (Supplementary Figures S4 and S5; and Supplementary File S8). Static ROC analyses showed some agreement (AUCs above 0.6) between RNA Framework reactivities for both *in silico* and RNA Framework informed ScanFold models at all *z*-score cutoffs (Supplementary Figure S8). Even though differences existed, most low z-score structures modeled by *in silico* ScanFold matched that of DMS-informed models, as shown for SARS-CoV-2 (59). Windowed ROC analysis (Supplementary File S9) demonstrated good agreement (high AUC values) for the majority of low z-score structures, and that low *z*-score regions with lower than expected AUC values correlated with dynamic regions identified by DRACO (Supplementary Figures S9-S10). Globally, low z-score structures found using *in silico* ScanFold were largely unaffected by inclusion of DMS reactivities, reflecting the stability of low z-score structures. This also reflects the ability of DMS to inform structural modeling while only causing minimal structure perturbation (78). Overall, this demonstrates the utility of ScanFold for identifying sequence-ordered, well-structured regions within mRNAs, making *in silico* predictions a valuable starting point. The addition of DMS data, however, provides critical information on the endogenous secondary structure of surrounding sequences, including longer-range interactions and structural dynamics–both of which cannot be addressed using ScanFold alone.

The low z-score regions identified in AR transcripts informed by DMS reactivities are well-structured and comprise higher probability base pairs. As expected, DMS reactivities at nucleotides with low average z-scores were generally lower than nucleotides with higher average *z*-scores (Supplementary Figure S6A). These nucleotides were not completely unreactive, though, revealing unpaired bulges and loops. As the *z*-scores increased (reflecting less bias toward ordered structure) the number and magnitude of DMS reactivities also increased due to chemical accessibility of nucleotides in less structured regions. Comparison of base pair probabilities (79) to RNA Framework informed *z*-scores demonstrated that nucleotides with lower mean *z*-scores had higher base pair probabilities, with the highest *z*-scores–predicted to be unpaired–showing the largest decrease in pairing probability (Supplementary Figure S6B).

### Dynamic RNA structural regions near to or overlapping ScanFold structures may attenuate function

Aside from being used to inform ScanFold, DMS reactivities generated from RNA Framework were also used to predict potential RNA structure heterogeneity using DRACO (54). DRACO extracted multiple reactivity profiles from the same dataset based on co-mutation events observed during the processing of DMS-MaPseq data. In the context of the surrounding static profile across the FL transcript, it was noted that in many instances the predicted structures were maintained while the surrounding sequence appeared to be dynamic. These subtle changes in structure are also representative of the evenly distributed stoichiometries for the conformations predicted by DRACO. For instance, the low MFE, low *z*-score and high covariation support seen in AR 6–7 (Figure 4; Supplementary Figure S11) supports the finding that highly ordered RNA structures have fewer changes in structure, potentially due to stronger evolutionary selection for functional conformations (80). Interestingly, the conformational changes that surround ScanFold structures may play roles in regulatory functions by mediating interactions (21,81). By altering the orientation, positioning, and spacing of the uniquely ordered structures their function may be fine-tuned at both the secondary and tertiary structure level (22). Additional work is needed to tease out the influence that flanking dynamic structure may have on the function of rigid local RNA structure.

For some dynamic regions, such as the CAG repeat region of the CDS and the A-rich structure AR 32 in the 3′UTR, parts of the ScanFold predicted structures varied as pairing patterns shifted between the repetitive sequence elements (Supplementary Figures S12 and S13). The CAG repeat region showed that both the static reactivity profile and one DRACO profile generated the same type of ‘frozen’ hairpin that is locked in by three CUG repeats as reported by de Mezer *et al.* (82). The remaining DRACO profiles generated alternative CAG–CUG pairings that formed multiple different hairpins. This supports an assertion that uninterrupted repeats can form slippery hairpins, whose length is reduced by pairing the repeat-specific flanking sequence (83). For structure 32, most of the hairpin consists of AU and GU pairs with many stretches of poly(A) repeats in the large terminal loop and AAAU repeats downstream. Perhaps these features allow the hairpin to ‘slip’ or form alternative conformations in cells. These changes could also be the result of different intra- or intermolecular interactions caused by differences in the mRNA life cycle, cell cycle, or response to internal and external stimuli (84). The structural changes also recapitulate some of the discrepancies in sliding window ROC data as well as differences seen between *in silico* ScanFold, informed ScanFold, and DRACO models (Supplementary Figure S4 and Supplementary File S8).

### Structured regions identified in AR-FL are biologically active *in vitro* and may associate with regulatory RNA binding proteins

Through our combined analysis of z-scores, covariation, interaction partners, and structural dynamics we were able to find several interesting structures in the AR-FL 5′ and 3′UTR. All tested structures showed evidence of function in reporter assays, but those found in the 5′UTR elicited the most dramatic results (Figures 4 and 5). When we tested AR 1–4, we noted that the large terminal-loop containing structure, AR 4, was sufficient to reduce protein and mRNA levels compared to vector control (Figure 4D). Within the terminal loop of the conserved hairpin (covariation for 6 of 7 base pairs), three separate consensus PCBP2 binding motifs were identified that matched those previously found in the AR-FL 3′UTR (30) and other human 3′UTRs such as tyrosine hydroxylase (85,86) and erythropoietin (87,88). We verified the interaction in the 5′UTR by RNA immunoprecipitation and by mass spectrometry of biotinylated RNA pulldowns from 22Rv1 lysates. Mutation of these binding motifs in AR 4 rescued protein and mRNA levels compared to WT control in our reporter assays. This novel interaction of PCBP2 with the AR 5′UTR is one of only a few examples of this RBP binding to a non-IRES region of a human 5′UTR (73,89,90) and possibly regulating the AR transcript through both UTRs (74). PCBP2 may be responsible for the changes seen in our assays as it has been shown to bind human UTRs (non-IRES regions) and effect mRNA stability (91,92), transcript turnover (93), and protein expression (73,90). Perhaps most interesting, is that PCBP2 interaction sites at either end of AR-FL have potentially opposing effects: destabilizing in the 5′UTR (Figure 4D) and stabilizing in the 3′UTR (30).

Downstream of AR 1–4, AR 6–7 is also able to significantly decrease protein and mRNA levels *in vitro* (Figure 4F). A notable feature of this region is a large A-rich stretch of 99 nt (AR 6–7 Int) with little propensity for forming secondary structure and no predicted base pairing. When AR 6–7 Int was tested, similar reductions in protein and mRNA levels were observed, but not quite to the extent as when both the flanking structures, AR 6 and 7, were included. This effect was consistent across all cell lines, but the significant drop in translational efficiency was only observed in the prostate cancer cell lines. Interestingly, the extent of translational inhibition was the same whether AR 6 and 7 were there or not, suggesting the primary site of translation regulation may be AR 6–7 Int, while flanking structure may play roles in mRNA stability and maintaining the accessibility of the single-stranded RNA region.

In the 3′UTR of AR-FL, AR 32 demonstrated a propensity to stabilize mRNA in HeLa cells, even with the additional inclusion of the nearby validated miR-297 and miR-9-5p binding sites in a larger construct (39,40,43). Similar stabilization was not observed in either 22Rv1 or DU145 cells, which may be explained by differences in regulatory proteins between cell lines. Indeed, the proteins that bound AR 32 during RNA pulldowns in 22Rv1 lysate are significantly enriched for functions in the regulation of mRNA stabilization and metabolism (Supplementary File S12) (94,95). Additionally, we found selective binding of the multi-functional protein CSDE1 in 22Rv1 lysate. Previous studies have shown that CSDE1 possesses multiple functions including activation and inhibition of translation, stabilization and destabilization of mRNA, and regulation of mitosis and apoptosis (96). Even though expression of this protein is higher in HeLa cells (71), the interaction and specific function of the protein is dependent on many factors including cell type (77), which may account for the decreased levels of reporter mRNA in prostate cancer cell lines compared to HeLa.

### Loss of RNA structures in the truncated AR-V7 isoform may have implications for AR gene deregulation

While a major driver of AR-V7 expression in prostate cancer is transcriptional activation, the significance of deregulation of post-transcriptional mechanisms (beyond splicing regulation) in AR-V7 is becoming more apparent (97). Relevant to this, the AR-FL structures that were found to have functional activity are missing in the AR-V7 isoform due to truncations of the 5′UTR and the total loss/replacement of the large FL 3′UTR (Figure 1). Indeed, only 13 highly stable AR-FL structures were retained in the shorter isoform. Lack of functional structure in AR-V7 could play a role in the expression of this prostate cancer associated transcript. These common and unique structures could also be potential sites for binding of small molecules/RIBOTACs that to reduce the expression of AR that drives prostate cancer progression (28,29). Comparing FL and V7 mRNA there were overall changes in structural propensity with average z-scores across each transcript of –0.58 and –0.35, respectively, which becomes more notable when partitioning results between the UTRs and coding sequences. Looking at the FL 5′UTR, 3′UTR and CDS *z*-scores are –0.66, –0.25 and –0.70 compared to –0.50, –0.13 and –0.62 for V7. Similar to the changes in structure between the isoforms, this change in propensity for formation of functional structures could play a role in the post-transcriptional regulation of AR-FL and V7. Similarly, RNA structural dynamics differ between AR-FL and AR-V7. Interestingly, two different V7 dynamic regions were identified across the 5′UTR-CDS junction whereas none were identified for FL. Although the known change in sequence can have large effect on regulation and stability, the structural differences may also be significant to AR-V7 expression as they can further affect RNA stability, decay, and trans-acting factors interactions. With this in mind, roles of secondary structure in the post-transcriptional regulatory environment of each isoform need to be considered in addition to differences in sequence.

## Conclusion

Through the experimental and computational analysis of AR, we have characterized the RNA secondary structural landscapes of two important isoforms and identified several potentially functional structures in the 5′ and 3′UTRs. Comparison of *in silico* and DMS-informed ScanFold results, provided further evidence that our program performs well with and without probing data. Analysis of structural dynamics identified both well structured (low *z*-score) and weakly or unstructured regions of AR-FL and V7 transcripts that can sample multiple conformations. We show that dynamic regions tend to maintain low *z*-score structures while sampling conformations using flanking sequence. Additionally, low *z*-score structures in the 5′UTR were identified as interaction partners for different trans-acting factors. We confirmed the interaction of PCBP2 with the conserved stem loop structure 4 in the 5′UTR of AR-FL and identified several other RBPs that may be post-transcriptionally regulating active regions of the AR-FL UTRs. Our study has provided new insights into the structural and functional features of the AR mRNA, providing a foundation to facilitate further research on this complex target and its potential applications in RNA therapeutics.

## Supplementary Material

gkae220_Supplemental_Files

## Data Availability

The data underlying this article are available in the article and in its online Supplementary material (gel image, files, figures, and tables) as well as on Zenodo and SRA (raw sequencing data), at 10.5281/zenodo.10180281 and https://www.ncbi.nlm.nih.gov/sra/PRJNA1048882.
