# Supplementary material for: The RNA secondary structure of androgen receptor-FL and V7 transcripts reveals novel regulatory regions
Source: Nucleic Acids Res. 2024 Mar 30;52(11):6596–613. doi: 10.1093/nar/gkae220 (PMC11194067; doi:10.1093/nar/gkae220)
Supplement: gkae220_Supplemental_Files [file gkae220_supplemental_files.zip › Combined_Supplementary_Revision.pdf]

Supplementary File S2

AR FL PCR Amplification Primers

AR FL V7 Span Fwd:
5’- CTAGCTGCACATTGCAAAGAAG-3’
AR FL Rev Seq 1:
5’-GGATCACTTCGCGCACGCTCT-3’

AR FL Fwd Seq 2:
5’-CCGCATCATCACAGCCTGTTGAACTCT-3’
AR FL Rev Seq 2:
5’-GTAGTCGCGACTCTGGTACGCAGC-3’

AR V7 Fwd Seq 3-2:
5’- GCGTTGGAGCATCTGAGTCCAGG-3’
AR FL Exon Test Rev 1:
5’-GTCCATACAACTGGCCTTCTTCGGC-3’

AR FL Exon Test Fwd 2:
5’-GCCAGGAAAGCGACTTCACCG-3’
AR FL Rev Seq 3:
5’-GAGCAAGGCTGCAAAGGAGTCGG-3

AR FL Fwd Seq 4:
5’-GGAGCCCGGAAGCTGAAGAAACTTG-3
AR FL Rev Seq 4:
5’-CACAACTTGACACTGGGCCATATGAGGA-3

AR FL Fwd Seq 5:
5’-CTGCAGTGCCTTGGGGAATTTCCTC-3’
AR FL Rev Seq 5:
5’-GTCAGTGTCCTAAGCAGGAGAACAGCC-3’

AR FL Fwd Seq 6:
5’-CAGCCAAAACTTGGCGACTTCCACAG-3’
AR FL Rev Seq 6:
5’-GGCAGAGGCCAAGATGAGAAAGTAGGTG-3’

AR FL Fwd Seq 7:
5’-GGAAAGGTCTGGTTGGTGTGGCTC-3’
AR FL Rev Seq 7:
5’-AGCACTGCCAACTTGTTTGGAGATGGT-3’

AR FL Fwd Seq 8:
5’-GCATGCGCTCTGCTCTACAAACAGAGT-3’
AR FL Rev Seq 8:
5’-TGGTGAGAACAGATCTGCCTACAAAGGCA-3’

AR FL Fwd Seq 9:
5’-CGAATTATCTTGTGCCAGTTGCCCAGGT-3’
AR FL Rev Seq 9:
5’-GGCCTTCCCTGTGGTAAATCTCAGTCAG-3’

AR FL Fwd Seq 10:
5’-CTATTGGGTTTGACCCACAGGTCCTGT-3’
AR FL Rev Seq 10:
5’-CTGATGGTGGCTGCTGTCAGCATC-3’

AR FL Fwd Seq 11:
5’-GCTGTTTCTCTGGTGGTTCCCTCTCTG-3’
AR FL Rev Seq 11:
5’-CGATCCTGCAGCCATAACAGGGGAT-3’

AR V7 PCR Amplification Primers

AR V7 Fwd Seq 1:
5’-GCGGAGAGAACCCTCTGTTTTCCC-3’
AR FL Rev Seq 1:
5’-GGATCACTTCGCGCACGCTCT-3’

AR FL Fwd Seq 2:
5’-CCGCATCATCACAGCCTGTTGAACTCT-3’
AR V7 Exon Test Rev 1:
5’-GTCCATACAACTGGCCTTCTTCGGC-3’

AR V7 Exon Test Fwd 2:
5’-GCCAGGAAAGCGACTTCACCG-3
AR V7 Rev Seq 3:
5’-AAACACTATTGGTCCCGCTGGAGGG-3’

AR V7 Fwd Seq 4:
5’-ACCCTGAAGAAAGGCTGACTTGCCTC-3
AR V7 Rev Seq 4:
5’-CTCCCCTCCTGAAGTCCTTCCTTGG-3’

Cloning Primers

GLO_5OL_Fwd:
5’-ACGAGCTCGCTAGCC-3’
GLO_3OL_Rev:
5’-CTGCAGGTCGACTCTAGAC-3’

RIP Primers

Yeap_et_al_3UTR_Fwd:
5’-ATGAACTTCGAATGAACTACATCAAGG-3’
Yeap_et_al_3UTR_Rev:
5’-GGAACATGTTCATGACAGACTGTAC-3’

AR_FL_5UTR_1-2_Fwd:
5’-GGTCCGGAGCAAGCCCAGA-3’
AR_FL_5UTR_1-2_Rev:
5’-CTGCAAGAGGCGTTGGCTGTC-3’

In vitro Transcription Primers

T7_GLOi_F:
5’-TAATACGACTCACTATAGGGACGAGCTCGCTAGCC-3’
GLO_3OL_R:
5’-CTGCAGGTCGACTCTAGAC-3’

Fwd_T7_M4_WT_SS:
5’-TAATACGACTCACTATAGGGCTTTGTCCTCCTCC-3’
Rev_T7_M4_WT_SS:
5’-CAGCTGTGGGAGAGAAGACGGG-3’

Fwd_T7_M4_Mut_SS:
5’-TAATACGACTCACTATAGGGCTTTGTCCTAATCCTCTCC-3’
Rev_T7_M4_Mut_SS:
5’-CAGCTGTGGGAGAGAAGACGGTTTAGG-3’

CT Files	In Silico vs RNA Framework Informed	In Silico vs Shapemapper2 Informed	RNA Framework Informed vs Shapemapper2 Informed	RNA Framework Informed Rep 1 vs Rep 2	RNA Framework Informed Rep 1 vs Rep 3	RNA Framework Informed Rep 2 vs Rep 3
**FL No Filter**	87.66%	83.64%	87.78%	88.26%	89.87%	87.04%
**FL -1**	94.00%	92.04%	93.90%	94.04%	95.86%	93.69%
**FL -2**	97.76%	97.33%	98.00%	98.17%	98.97%	98.27%
**V7 No Filter**	78.67%	77.01%	80.89%	88.07%	89.41%	85.62%
**V7 -1**	90.59%	92.31%	91.09%	96.82%	96.76%	96.74%
**V7 -2**	96.68%	98.87%	96.90%	98.95%	98.81%	99.56%

Supplementary Table S1. Per nucleotide comparison of base percent pairing consistency. Result are for the no filter, -1, and -2 z-score cutoff comparing in silico ScanFold to RNA Framework informed ScanFold and Shapemapper2 informed ScanFold as well as comparison of RNA Framework informed ScanFold replicates.

Tool/ Algorithm	Link	Description
ScanFold	https://mosslabtools.bb.iastate.edu/	ScanFold is an RNA sequence scanning pipeline that deduces local structural stability, propensity for unusual sequence-ordered stability, and likely functional secondary structures by generating consensus structures where base pairs are weighted by their contribution to ordered structural stability. ScanFold uses a sliding window to analyze the entire mRNA sequence of interest. The sequence of each window is folded via RNAfold to calculate its native minimum free energy (MFE) and associated secondary structure. The native sequence in each window is shuffled and folded 100 times to calculate an average randomized MFE. The native and average randomized MFE values are then used to calculate the thermodynamic z-score. The z-scores are then analyzed to generate a consensus secondary structure model based on paired nucleotides that recur across low z-score analysis windows.
RNA Framework	https://rnaframework-docs.readthedocs.io/en/latest/	RNA Framework is an all-in-one suite of programs that was developed to process RNA structure probing and post-transcriptional modifications data generated by sequencing. Features include indexing, mapping, mutation counting, reactivity normalization, reactivity informed MFE folding, calibration of folding parameters, structure extraction, structural comparisons, reactivity correlations, generation of

		analysis files, and generation of input files for DRACO.
DRACO	https://github.com/dincarnato/draco	Using a combination of spectral deconvolution and fuzzy clustering, DRACO can deconvolute RNA structural heterogeneity in a biological sample. With the capability of mutational profiling (MaP) experiments, DMS or SHAPE, to introduce multiple mutations on a single cDNA, DRACO can extract multiple reactivity profiles from the same dataset allowing for identification of potentially dynamic RNA secondary structures and reconstruction of their relative stoichiometries.
Shapemapper 2	https://github.com/Weeks-UNC/shapemapper2	ShapeMapper2 is a tool developed to process RNA structure probing data from mutational profiling (MaP) experiments. It performs automated calculation of RNA chemical probing reactivities from the MaP experiments, where the modifications to the RNA are detected as mutations in cDNA after reverse transcription and high throughput sequencing. ShapeMapper2 is broadly useful for many types of probing experiments including SHAPE and DMS.
SuperFold	https://github.com/Weeks-UNC/Superfold	SuperFold is a tool that takes the output data from ShapeMapper2 to model RNA secondary structures. Folding is done using RNAStructure and can identfify pseudoknots; identify de novo regions with stable structures; and visualize most probable helices, alternative helices, base pair probabilities, and shannon entropies.

CM-Builder	https://github.com/dincarnato/labtools/tree/master	Using a single sequence with or without a secondary structural model, cm-builder automatically builds and refines covariation models (CMs). This program uses Infernal to generate CMs from a single sequence and structure before iteratively refining the model by searching against a user generated BLAST database of related sequences.
R-scape/ CaCoFold	http://eddylab.org/R-scape/	Using the output of cm-builder, R-scape/CaCoFold performs statistical tests to identify conserved RNA structure. R-scape measures pairwise covariations events observed in the alignment file from cm-builder and analyzes all possible pairs, including those in the user's predicted structure if it is provided. To eliminate false positive covariation events. R-scape uses a null hypothesis that takes phylogenetic correlations and base composition biases into account. R-scape will calculate covariation using available secondary structure data, and it can calculate a structure based on the observed covariations using CaCoFold.
RNAfold	https://www.tbi.univie.ac.at/RNA/RNAfold.1.html	This program uses the Turner Rules to calculate the minimum free energy (MFE) secondary structures and partition function of any input RNA sequence. RNAfold also allows for the use of many flags such as incorporation of reactivity data and the use of max pairing distances.

VARNA	https://varna.lisn.upsaclay.fr/	VARNA is Java Applet designed to draw RNA secondary structure. VARNA is a user friendly that allows the user to either copy and paste the sequence and structure (in dot bracket notation) into the program or drag and drop a .CT file. VARNA allows for modification of almost all components of the structure including the addition of "heat maps" that can display many types of data, the type of base pair, the orientation of all structure, annotation of regions, and much more.
Integrative Genomics Viewer (IGV)	https://www.igv.org/	IGV is a user friendly, high-performance tool for the visualization and exploration of genomic data. It can be download and ran as a desktop application or used as a web based application. This is an interactive tool that supports a wide variety of genomic data and file types. It allows users to upload many different tracks, modify these tracks, and export data for figure generation.

Supplementary Table S2. All algorithms and bioinformatics tools used in this study. The name, link to the developer's page, and description of each program can be found in the columns moving left to right.

	RNAFramework Merged Datasets				Shapemapper2 Merged Datasets		
**Transcript**	**Average Reactivity**	**Number of Reads**	**Read Coverage**	**Mean Read Depth**	**Average Reactivity**	**Average Coverage ≥ 5000 Reads**	**Highly Reactive Nucleotides**
Long (FL)	0.428	8789608	87.80%	119317	0.405	84.20%	34.1%
Short (V7)	0.480	3401494	95.70%	130355	0.422	90.50%	37.1%

Supplementary Table S3. Sequencing statistics obtained from processing merged data using RNA Framework and Shapemapper2. Both methods resulted in similar average read coverage and average DMS reactivity.

Transcript Replicate	Average Reactivity	Number of Reads	Read Coverage	Mean Read Depth
FL Rep 1	0.359	3317970	86.99%	45186.5
FL Rep 2	0.290	2880942	87.00%	38923.7
FL Rep 3	0.364	2590696	87.18%	35207.0
V7 Rep 1	0.416	1014652	93.36%	39781.4
V7 Rep 2	0.353	1518714	92.86%	56409.0
V7 Rep 3	0.434	868128	95.55%	34144.5

Supplementary Table S4. Sequencing statistics obtained from processing individual datasets using RNA Framework. All replicates for the respective isoform resulted in similar numbers of reads, average coverage, mean read depth, and average reactivity.

Method	Transcript	GC%	ΔG (MFE)	ED	Average ZS	Windows	ZS <-1	ZS <-2	Total BP	BP ZS <-1	BP ZS <-2	Motifs
In Silico ScanFold	Long (FL)	48.10	-28.48	25.74	-0.58	10548	33.41%	10.65%	42320	33.68%	9.48%	27
RNA Framework Informed		48.10	-27.71	25.74	-0.58	10548	33.13%	10.55%	39538	35.28%	8.92%	26
In Silico ScanFold	Short (V7)	55.76	-34.23	27.47	-0.35	3496	26.69%	5.89%	16220	23.79%	4.61%	6
RNA Framework Informed		55.76	-32.06	27.47	-0.35	3496	26.86%	5.49%	15278	25.04%	4.50%	5
In Silico ScanFold	Long (FL)	48.10	-28.48	25.74	-0.58	10548	33.41%	10.65%	42320	33.68%	9.48%	27
Shapemapper2 Informed		48.10	-30.73	25.74	-0.58	10548	33.09%	10.55%	40878	35.61%	9.64%	27
In Silico ScanFold	Short (V7)	55.76	-34.23	27.47	-0.35	3496	26.69%	5.89%	16220	23.79%	4.61%	6
Shapemapper2 Informed		55.76	-36.08	27.47	-0.34	3496	26.12%	5.95%	15154	25.80%	5.03%	4

Supplementary Table S5. ScanFold metrics from *in silico* vs RNA Framework informed ScanFold and in silico vs Shapemapper2 informed ScanFold of AR-FL and V7. All metrics were generated using default ScanFold parameters and obtained or calculated from various output files.

	Z-score	ScanFold Reference Model	ScanFold Predicted Model	PPV	Sensitivity
**FL**	-2	RNA Framework Informed	In Silico	0.644	0.691
	-1	RNA Framework Informed	In Silico	0.798	0.844
	No Filter	RNA Framework Informed	In Silico	0.781	0.802
	-2	Shapemapper2 Informed	In Silico	0.587	0.609
	-1	Shapemapper2 Informed	In Silico	0.710	0.798
	No Filter	Shapemapper2 Informed	In Silico	0.700	0.740
	-2	RNA Framework Informed	Shapemapper2 Informed	0.710	0.735
	-1	RNA Framework Informed	Shapemapper2 Informed	0.844	0.794
	No Filter	RNA Framework Informed	Shapemapper2 Informed	0.809	0.785
**V7**	-2	RNA Framework Informed	In Silico	0.080	0.075
	-1	RNA Framework Informed	In Silico	0.556	0.669
	No Filter	RNA Framework Informed	In Silico	0.601	0.645
	-2	Shapemapper2 Informed	In Silico	0.780	0.780
	-1	Shapemapper2 Informed	In Silico	0.662	0.760
	No Filter	Shapemapper2 Informed	In Silico	0.620	0.619
	-2	RNA Framework Informed	Shapemapper2 Informed	0.160	0.150
	-1	RNA Framework Informed	Shapemapper2 Informed	0.559	0.586
	No Filter	RNA Framework Informed	Shapemapper2 Informed	0.648	0.695

Supplementary Table S6. PPV and Sensitivity analysis results for FL and V7 transcripts. Comparisons were completed using RNA Framework informed, Shapemapper2 informed, and in silico models.

FL		
**CT**	**React**	**AUC**
FL_in_silico_Zavg_NoFilter.ct	RNAFramework_Merged_FL.react	0.627
FL_in_silico_Zavg_-1_pairs.ct	RNAFramework_Merged_FL.react	0.640
FL_in_silico_Zavg_-2_pairs.ct	RNAFramework_Merged_FL.react	0.654
FL_RNAFramework_Informed_Zavg_NoFilter.ct	RNAFramework_Merged_FL.react	0.655
FL_RNAFramework_Informed_Zavg_-1_pairs.ct	RNAFramework_Merged_FL.react	0.659
FL_RNAFramework_Informed_Zavg_-2_pairs.ct	RNAFramework_Merged_FL.react	0.650
FL_in_silico_Zavg_NoFilter.ct	Shapemapper2_FL.react	0.607
FL_in_silico_Zavg_-1_pairs.ct	Shapemapper2_FL.react	0.598
FL_in_silico_Zavg_-2_pairs.ct	Shapemapper2_FL.react	0.579
FL_Shapemapper2_Informed_Zavg_NoFilter.ct	Shapemapper2_FL.react	0.615
FL_Shapemapper2_Informed _Zavg_-1_pairs.ct	Shapemapper2_FL.react	0.604
FL_Shapemapper2_Informed _Zavg_-2_pairs.ct	Shapemapper2_FL.react	0.581
FL_Merged_RNAFramework_120.ct	Shapemapper2_FL.react	0.659
FL_SuperFold_120.ct	RNAFramework_Merged_FL.react	0.708
FL_RNAFramework_Informed_Zavg_NoFilter.ct	Shapemapper2_FL.react	0.610
FL_RNAFramework_Informed_Zavg_-1_pairs.ct	Shapemapper2_FL.react	0.603
FL_RNAFramework_Informed_Zavg_-2_pairs.ct	Shapemapper2_FL.react	0.568
FL_Shapemapper2_Informed_Zavg_NoFilter.ct	RNAFramework_Merged_FL.react	0.660
FL_Shapemapper2_Informed_Zavg_-1_pairs.ct	RNAFramework_Merged_FL.react	0.668
FL_Shapemapper2_Informed_Zavg_-2_pairs.ct	RNAFramework_Merged_FL.react	0.658

Supplementary Table S7. AR-FL area under the curve (AUC) values from static ROC analysis of in silico ScanFold, informed ScanFold and MFE predictions against merged RNA Framework and Shapemapper2 reactivity values

V7		
**CT**	**React**	**AUC**
V7_in_silico_Zavg_NoFilter.ct	RNAFramework_Merged_V7.react	0.631
V7_in_silico_Zavg_-1.ct	RNAFramework_Merged_V7.react	0.627
V7_in_silico_Zavg_-2.ct	RNAFramework_Merged_V7.react	0.615
V7_RNAFramework_Informed_Zavg_NoFilter.ct	RNAFramework_Merged_V7.react	0.688
V7_RNAFramework_Informed_Zavg_-1_pairs.ct	RNAFramework_Merged_V7.react	0.710
V7_RNAFramework_Informed_Zavg_-2_pairs.ct	RNAFramework_Merged_V7.react	0.680
V7_in_silico_Zavg_NoFilter.ct	Shapemapper2_V7.react	0.608
V7_in_silico_Zavg_-1.ct	Shapemapper2_V7.react	0.562
V7_in_silico_Zavg_-2.ct	Shapemapper2_V7.react	0.522
V7_Shapemapper2_Informed_Zavg_NoFilter.ct	Shapemapper2_V7.react	0.635
V7_Shapemapper2_Informed_Zavg_-1_pairs.ct	Shapemapper2_V7.react	0.578
V7_Shapemapper2_Informed_Zavg_-2_pairs.ct	Shapemapper2_V7.react	0.520
V7_Merged_RNAFramework_120.ct	Shapemapper2_V7.react	0.679
V7_SuperFold_120.ct	RNAFramework_Merged_V7.react	0.757
V7_RNAFramework_Informed_Zavg_NoFilter.ct	Shapemapper2_V7.react	0.625
V7_RNAFramework_Informed_Zavg_-1_pairs.ct	Shapemapper2_V7.react	0.589
V7_RNAFramework_Informed_Zavg_-2_pairs.ct	Shapemapper2_V7.react	0.524
V7_Shapemapper2_Informed_Zavg_NoFilter.ct	RNAFramework_Merged_V7.react	0.668
V7_Shapemapper2_Informed_Zavg_-1_pairs.ct	RNAFramework_Merged_V7.react	0.661
V7_Shapemapper2_Informed_Zavg_-2_pairs.ct	RNAFramework_Merged_V7.react	0.636

Supplementary Table S8. AR-V7 area under the curve values from static ROC analysis of in silico ScanFold, informed ScanFold and MFE predictions against merged RNA Framework and Shapemapper2 reactivity values

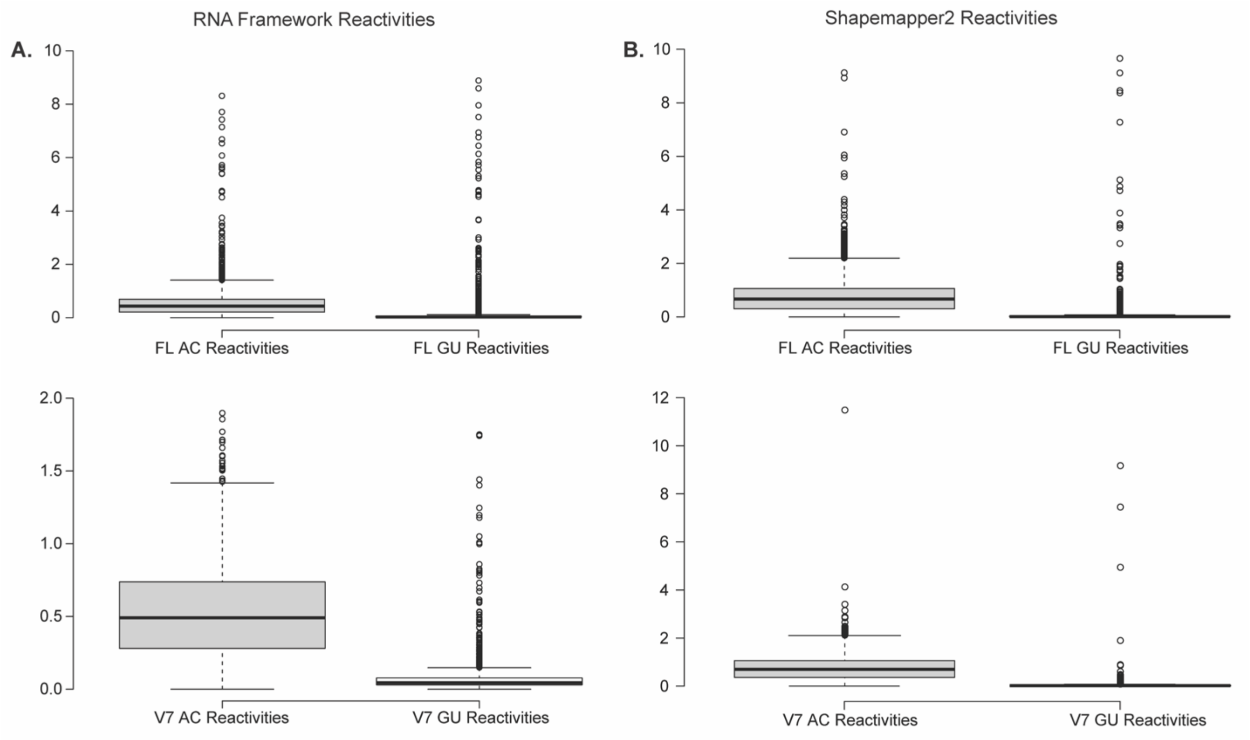

Supplementary Figure S1. Box plots of AR-FL and V7 reactivities normalized using RNA Framework and Shapemapper2. **A)** RNA Framework normalized reactivities for the FL (top) and V7 (bottom) transcripts. **B)** Shapemapper2 normalized reactivities for the FL (top) and V7 (bottom) transcripts. In all cases the A and C nucleotides were more reactive that G and U nucleotides, showing the specificity of DMS for direct probing of specific nucleotides.

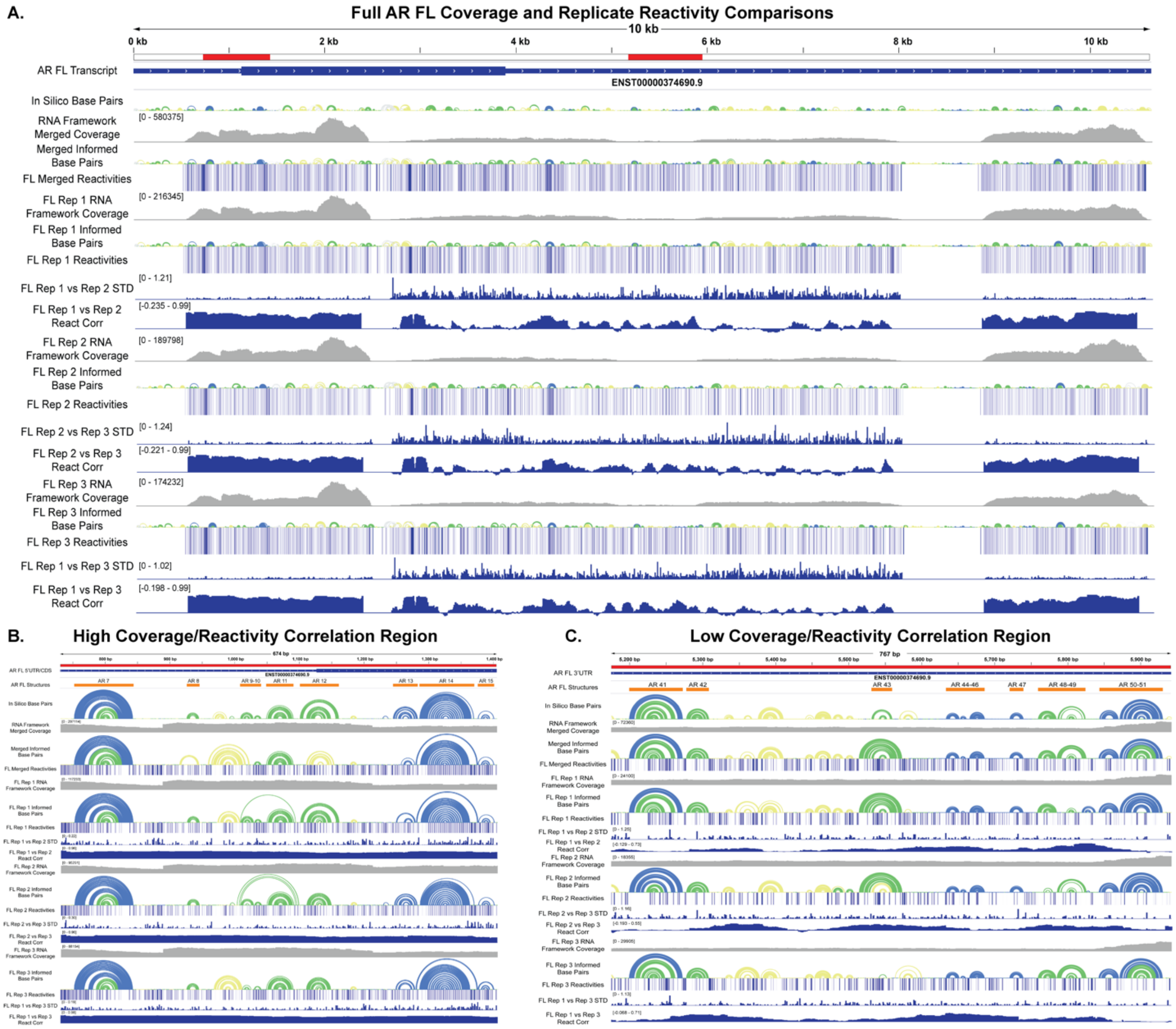

Supplementary Figure S2. AR-FL coverage and replicate reactivity comparisons. All data from merged and individual sequencing analysis are overlayed against in silico ScanFold data, 100nt sliding window Pearson correlation data, and per nucleotide reactivity standard deviation data. **A**) AR-FL transcript showing data for in silico base pairs, merged RNA Framework coverage data, merged RNA Framework informed ScanFold base pairs, merged RNA Framework reactivities, replicate 1 RNA Framework coverage data, replicate 1 RNA Framework informed ScanFold base pairs, replicate 1 RNA Framework reactivities, replicate 1 vs 2 per nucleotide reactivity standard deviation, replicate 1 vs 2 100nt sliding window Pearson correlation, replicate 2 RNA Framework coverage data, replicate 2 RNA Framework informed ScanFold base pairs, replicate 2 RNA Framework reactivities, replicate 2 vs 3 per nucleotide reactivity standard deviation, replicate 2 vs 3 100nt sliding window Pearson correlation, replicate 3 RNA Framework coverage data, replicate 3 RNA Framework informed ScanFold base pairs, replicate 3 RNA Framework reactivities, replicate 1 vs 3 per nucleotide reactivity standard deviation, and replicate 1 vs 3 100nt sliding window Pearson correlation. These data show a positive correlation between high coverage regions and both low standard deviation and high correlation. **B**) Here, a zoomed in view of the same data from panel A is shown for the region encompassing structures 7-15 in the 5'UTR and CDS. These data show that in a region with high coverage regions, low standard deviation, and high correlation the structure predictions are not significantly affected. **C**) Here, a zoomed in view of the same data from panel A is shown for the region encompassing structures 41-51 in the 3'UTR. These data show

that in a region with low coverage regions, high standard deviation, and low correlation the structure predictions still are not significantly affected. In total, the data shown here provides evidence that there are no PCR bottlenecks or bias in our approach. It also demonstrates that although overall replicate reactivity correlation is low, there are regions of high correlation and regardless of the correlation structure predictions are not significantly affected.

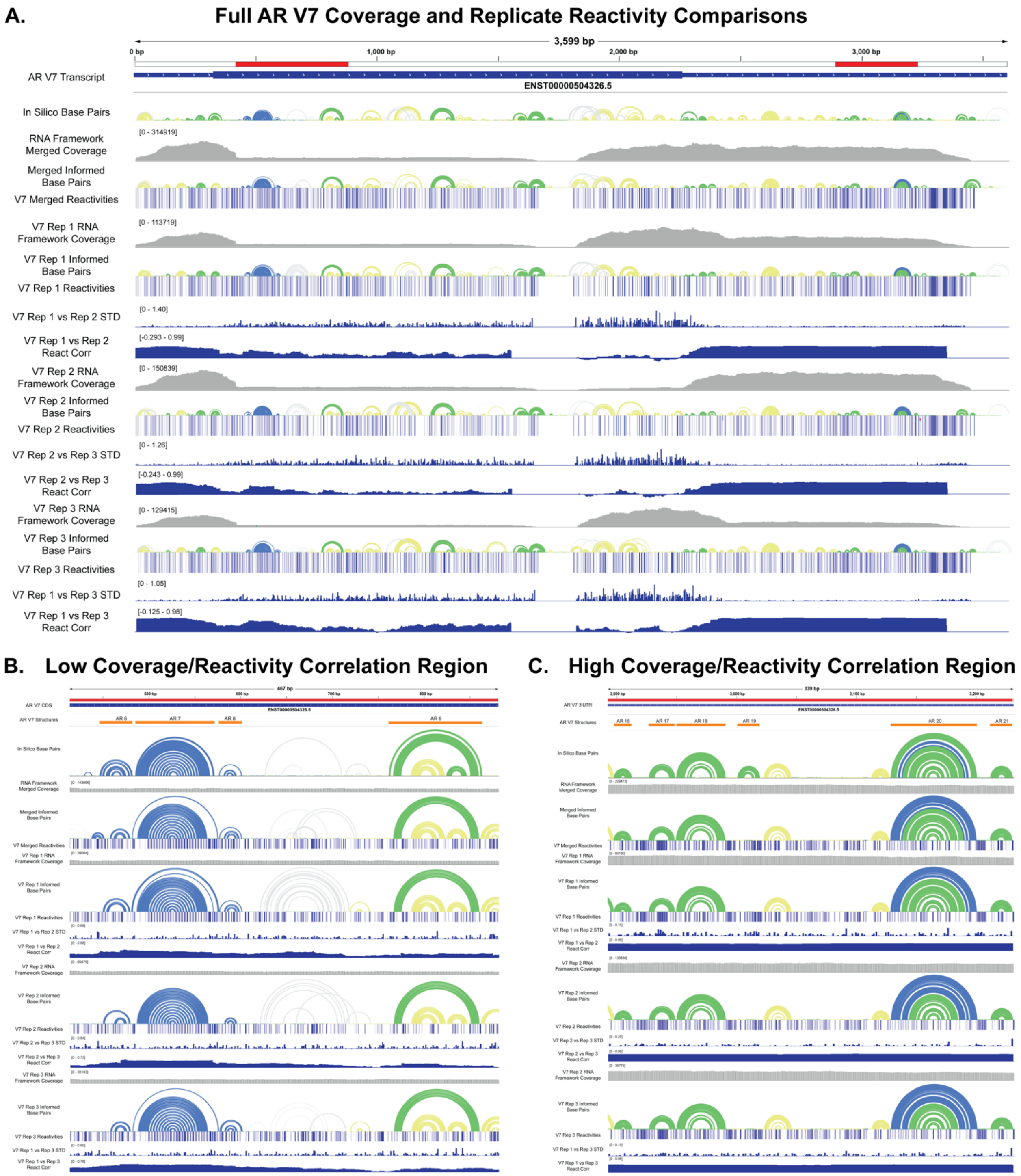

Supplementary Figure S3. AR-V7 coverage and replicate reactivity comparisons. All data from merged and individual sequencing analysis are overlayed against in silico ScanFold data, 100nt sliding window Pearson correlation data, and per nucleotide reactivity standard deviation data. **A**) AR-V7 transcript showing data for in silico base pairs, merged RNA Framework coverage data, merged RNA Framework informed ScanFold base pairs, merged RNA Framework reactivities, replicate 1 RNA Framework coverage data, replicate 1 RNA Framework informed ScanFold base pairs, replicate 1 RNA Framework reactivities, replicate 1 vs 2 per nucleotide reactivity standard deviation, replicate 1 vs 2 100nt sliding window Pearson

correlation, replicate 2 RNA Framework coverage data, replicate 2 RNA Framework informed ScanFold base pairs, replicate 2 RNA Framework reactivities, replicate 2 vs 3 per nucleotide reactivity standard deviation, replicate 2 vs 3 100nt sliding window Pearson correlation, replicate 3 RNA Framework coverage data, replicate 3 RNA Framework informed ScanFold base pairs, replicate 3 RNA Framework reactivities, replicate 1 vs 3 per nucleotide reactivity standard deviation, and replicate 1 vs 3 100nt sliding window Pearson correlation. These data show a positive correlation between high coverage regions and both low standard deviation and high correlation. **B**) Here, a zoomed in view of the same data from panel A is shown for the region encompassing structures 6-9. These data show that in a region with high coverage regions, low standard deviation, and high correlation the structure predictions are not significantly affected. **C**) Here, a zoomed in view of the same data from panel A is shown for the region encompassing structures 16-21 These data show that in a region with low coverage regions, high standard deviation, and low correlation the structure predictions still are not significantly affected. In total, the data shown here provides evidence that there are no PCR bottlenecks or bias in our approach. It also demonstrates that although overall replicate reactivity correlation is low, there are regions of high correlation and regardless of the correlation structure predictions are not significantly affected.

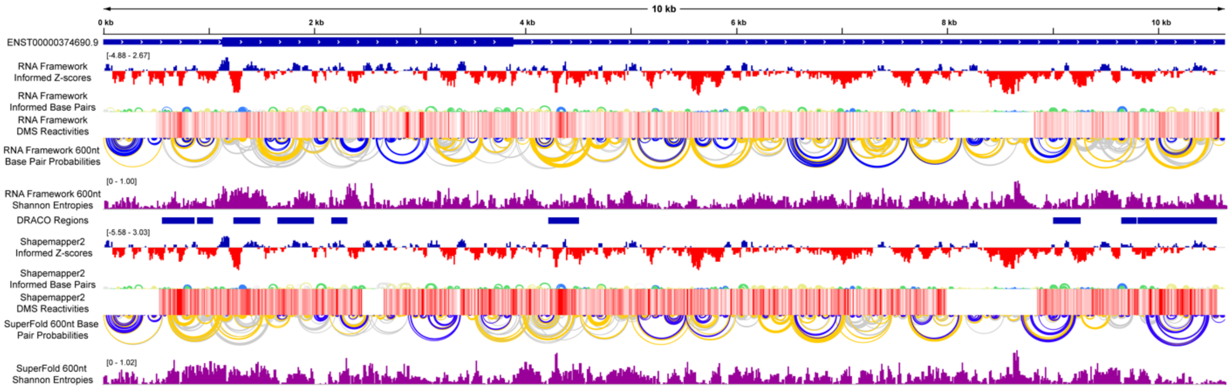

Supplementary Figure S4. RNA Framework and Shapemapper2/SuperFold informed prediction data for AR-FL. All data tracks are presented as visualized in the Integrative Genomics Viewer (IGV). Below the transcript cartoon are the RNA Framework informed ScanFold z-scores with positive values in blue and negative values in red. The predicted base pairs are represented as an arc diagram with z-score >0, >-1, >-2, and <-2 color in white/gray, yellow, green, and blue respectively. DMS reactivities are represented as a heat map with a scale of 0-1 where 0 is white, 1 is dark red, and intermediate values as shades of red. The base pair probabilities were calculated using a 600 nt maximum base pair span, and they are also represented as an arc diagram with probabilities >80%, 30-80%, and 10-30% shown in blue, gold, and gray respectively. The Shannon entropies were calculated using a 600 nt maximum base pair span and are represented as a purple bar graph. DRACO identified dynamic regions are shown with blue boxes below the Shannon entropy plot. Below the RNA Framework data, the same tracks are represented for Shapemapper2/SuperFold data. Comparison of the methods demonstrates minimal changes between the informed structure predictions. The low z-score structures also generally correspond with highly probable base pairings and the Shannon entropies, calculated using a 600 nt maximum base pair span, correspond to the base pair probabilities (i.e., high probabilities correspond to low entropy).

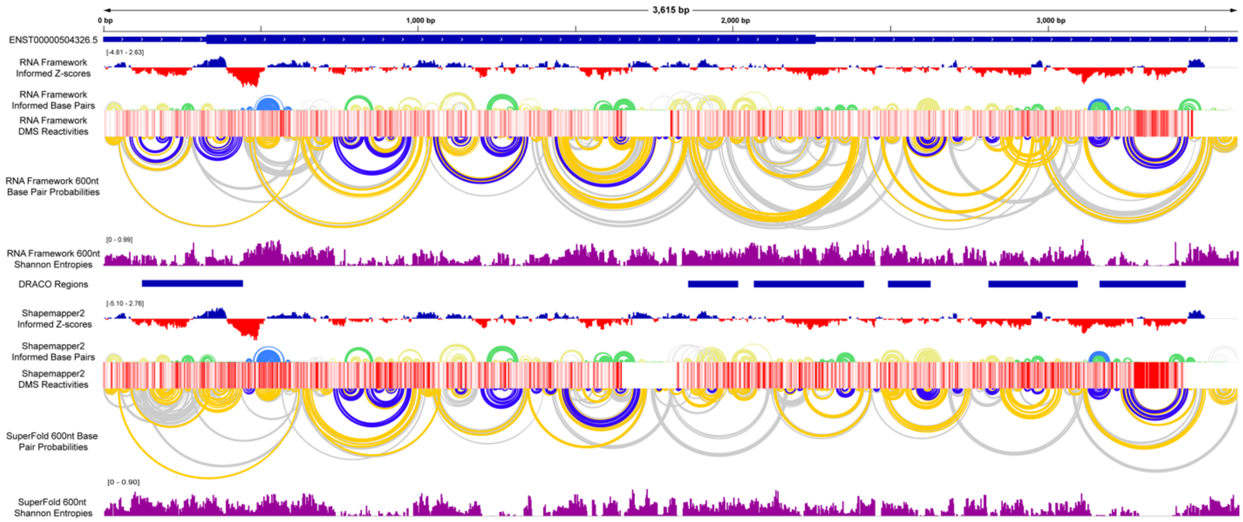

Figure S5. RNA Framework and Shapemapper2/SuperFold informed prediction data for AR-V7. All data tracks are presented as visualized in the Integrative Genomics Viewer (IGV). Below the transcript cartoon are the RNA Framework informed ScanFold z-scores with positive values in blue and negative values in red. The predicted base pairs are represented as an arc diagram with z-score >0, >-1, >-2, and <-2 color in white/gray, yellow, green, and blue respectively. DMS reactivities are represented as a heat map with a scale of 0-1 where 0 is white, 1 is dark red, and intermediate values as shades of red. The base pair probabilities were calculated using a 600 nt maximum base pair span, and they are also represented as an arc diagram with probabilities >80%, 30-80%, and 10-30% shown in blue, gold, and gray respectively. The Shannon entropies were calculated using a 600 nt maximum base pair span and are represented as a purple bar graph. DRACO identified dynamic regions are shown with blue boxes below the Shannon entropy plot. Below the RNA Framework data, the same tracks are represented for Shapemapper2/SuperFold data. Comparison of the methods demonstrates minimal changes between the informed structure predictions. The low z-score structures also generally correspond with highly probable base pairings and the Shannon entropies, calculated using a 600 nt maximum base pair span, correspond to the base pair probabilities (i.e., high probabilities correspond to low entropy).

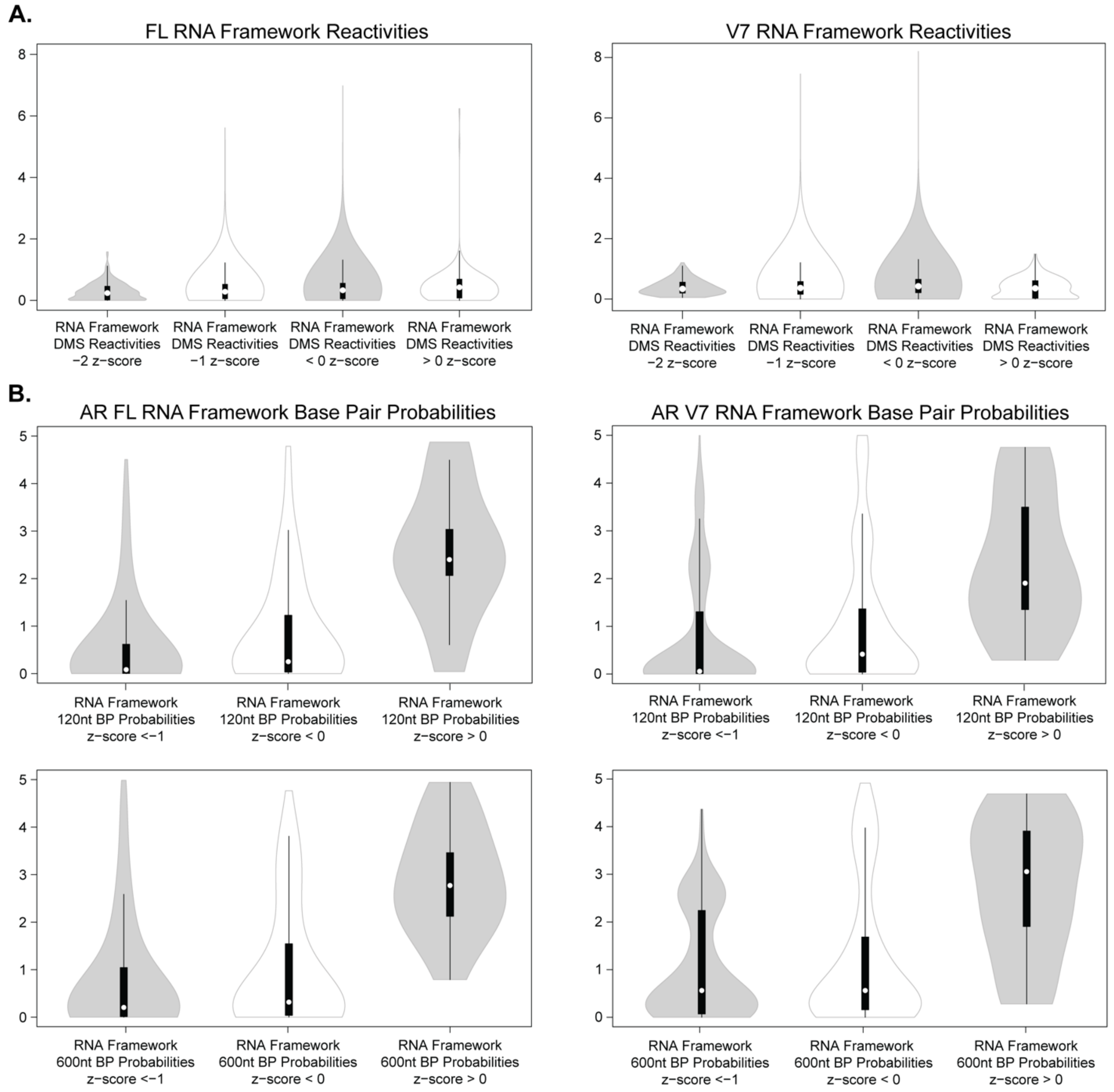

Figure S6. RNA Framework reactivites and base pair probabilities of nucleotides involved in ScanFold predicted structures. **A)** AR-FL RNA Framework reactivities binned based on z-scores of <-2, <-1, <0, and >0. Reactivities are the greatest for higher z-score structure as they are less stable and structured. **B)** AR-V7 RNA Framework base pair probabilities using 120 nt and 600 nt max base pair span binned based on z-scores of <-1, <0, and >0. In all cases lower z-scores result in higher base pair probabilities. ScanFold results are supported as lower z-score structures are more likely to form based on their uniquely ordered and stable nature.

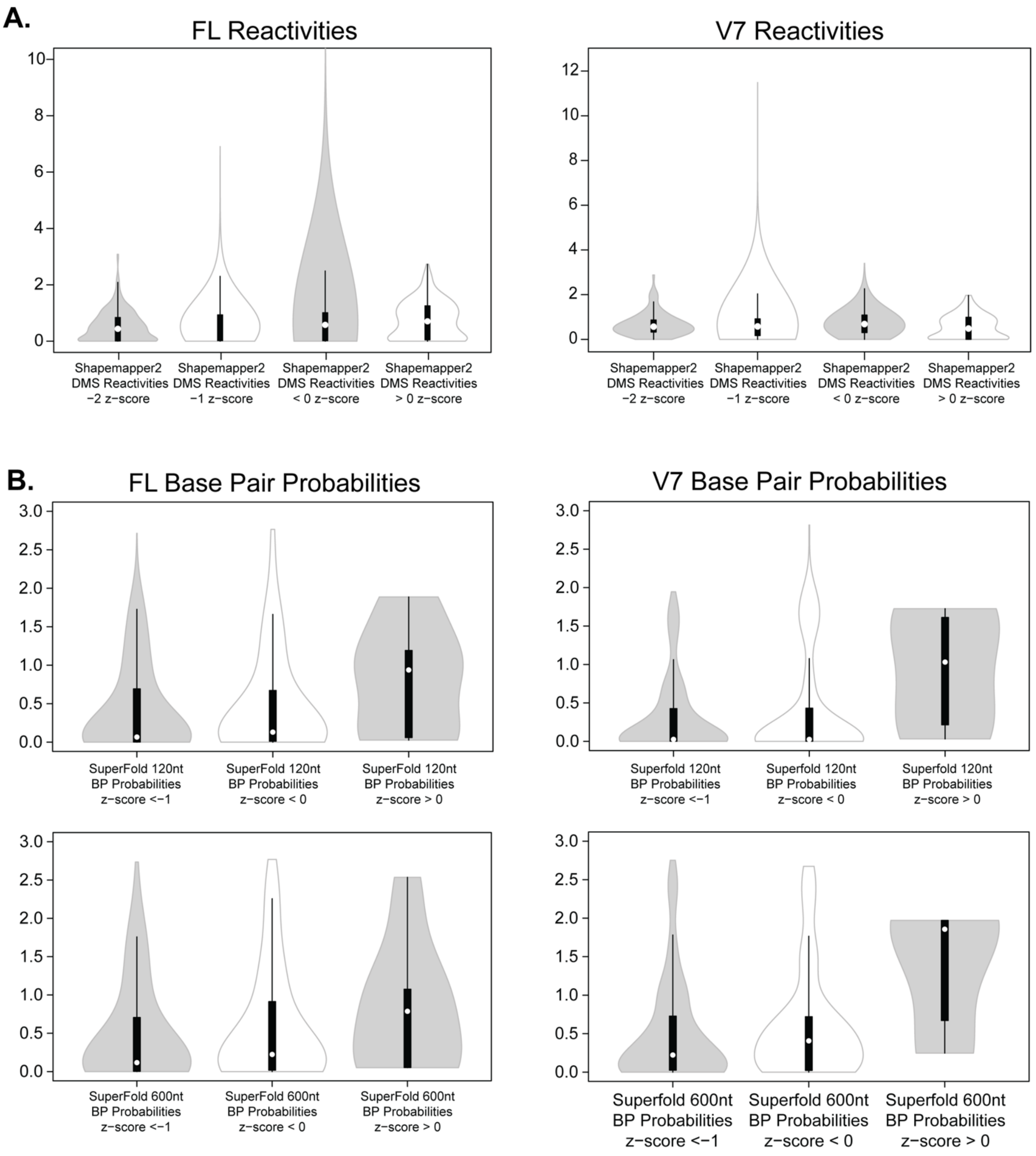

Figure S7. Shapemapper2 reactivites and SuperFold base pair probabilities of nucleotides involved in ScanFold predicted structures. **A)** AR-FL Shapemapper reactivities binned based on z-scores of <-2, <-1, <0, and >0. Reactivities are the greatest for higher z-score structure as they are less stable and structured. **B)** AR-V7 SuperFold base pair probabilities using 120 nt and 600 nt max base pair span binned based on z-scores of <-1, <0, and >0. In all cases lower z-scores result in higher base pair probabilities. ScanFold results are supported as lower z-score structures are more likely to form based on their uniquely ordered and stable nature.

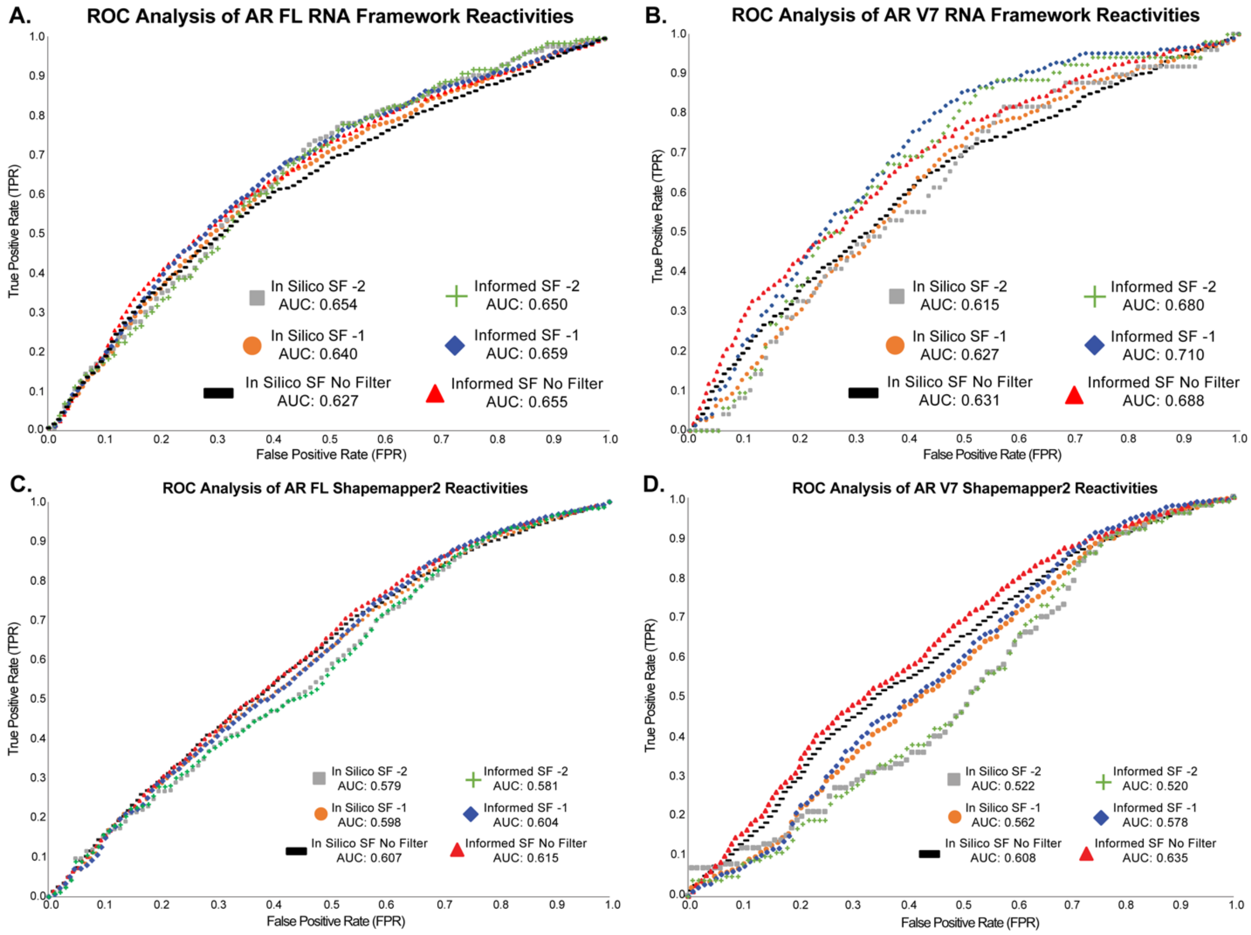

Figure S8. ROC analysis of AR-FL and V7 RNA Framework reactivities

ROC analysis of AR-FL and V7 RNA Framework and Shapemapper2 reactivities. ROC analysis was used to assess how well structural models agree with DMS reactivity data. **A**) AR-FL ROC analysis of the RNA Framework reactivities vs in silico ScanFold and RNA Framework informed ScanFold models at -2, -1, and no filter z-score cutoffs. All AUC values for both in silico and informed models were above 0.6, indicating general agreement between both models and reactivities at all z-score cutoffs. In the case of -1 and no filter z-scores, informed predictions performed slightly better than purely in silico models. **B**) AR-V7 ROC analysis of the RNA Framework reactivities vs in silico ScanFold and RNA Framework informed ScanFold models at -2, -1, and no filter z-score cutoffs. All AUC values for both in silico and informed models were above 0.6, indicating some agreement between models and reactivities. At all z-score cutoffs, informed predictions performed better than purely in silico models. **C**) ROC analysis of the AR-FL transcript in silico ScanFold and Shapemapper2 informed ScanFold models at -2, -1, and no filter z-score cutoffs. All AUC values for both in silico and informed models were near 0.6, indicating some agreement between both models and reactivities at all z-score cutoffs. At all z-score cutoffs, the informed models performed slightly better than purely in silico models. **D)** ROC analysis of the AR-V7 transcript in silico ScanFold and Shapemapper2 informed ScanFold models at -2, -1, and no filter z-score cutoffs. All AUC values for both in silico and informed models, except no filter, were below 0.6. For V7 the Shapemapper2 reactivities did not agree as well with in silico and informed ScanFold predictions although comparison of predictions showed minimal changes. In panel A-D in silico ScanFold models at -2, -1, and no filter z-score cutoff are represented as gray squares, orange circles, and black rectangles respectively. The RNA Framework and Shapemapper2 informed ScanFold models at -2, -1, and no filter z-score cutoffs are represented as green crosses, blue diamonds, and red triangles respectively.

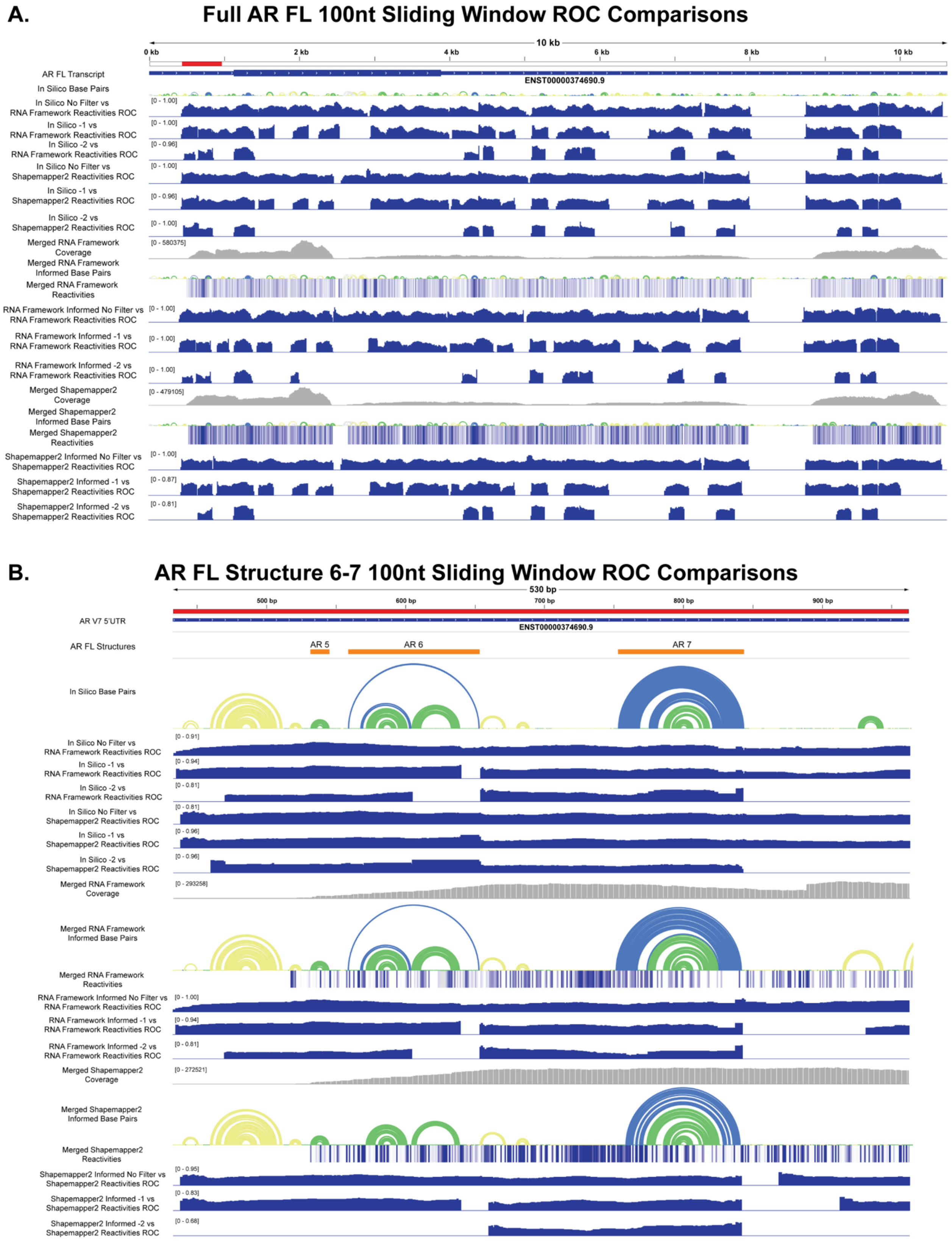

Figure S9. AR-FL 100nt sliding window ROC comparisons. All data from merged and individual sequencing analyses are overlayed against in silico ScanFold data, and 100nt sliding window ROC data. **A**) AR-FL

transcript showing data for in silico ScanFold base pairs; in silico ScanFold no filter, -1, -2 z-score structures vs RNA Framework reactivities ROC data; in silico ScanFold no filter, -1, -2 z-score structures vs Shapemapper2 reactivities; merged RNA Framework coverage; merged RNA Framework informed ScanFold base pairs; merged RNA Framework reactivities; merged RNA Framework informed ScanFold no filter, -1, -2 z-score structures vs RNA Framework reactivities ROC data; merged Shapemapper2 coverage; merged Shapemapper2 informed ScanFold base pairs; merged Shapemapper2 reactivities; and merged Shapemapper2 informed ScanFold no filter, -1, -2 z-score structures vs Shapemapper2 reactivities ROC data. This data shows regions of high and low AUC values across the transcript. High values generally correspond to low z-score structures and low values generally correspond to high z-score structure. **B**) All of the same data is presented here with a zoomed in look at AR-FL structure 6-7 in the 5'UTR. Although AUC values in this region are higher than the average across the transcript, we would expect them to be higher than they are. This discrepancy agree with the DRACO results indicating structural heterogeneity and potential structural dynamics in this region.

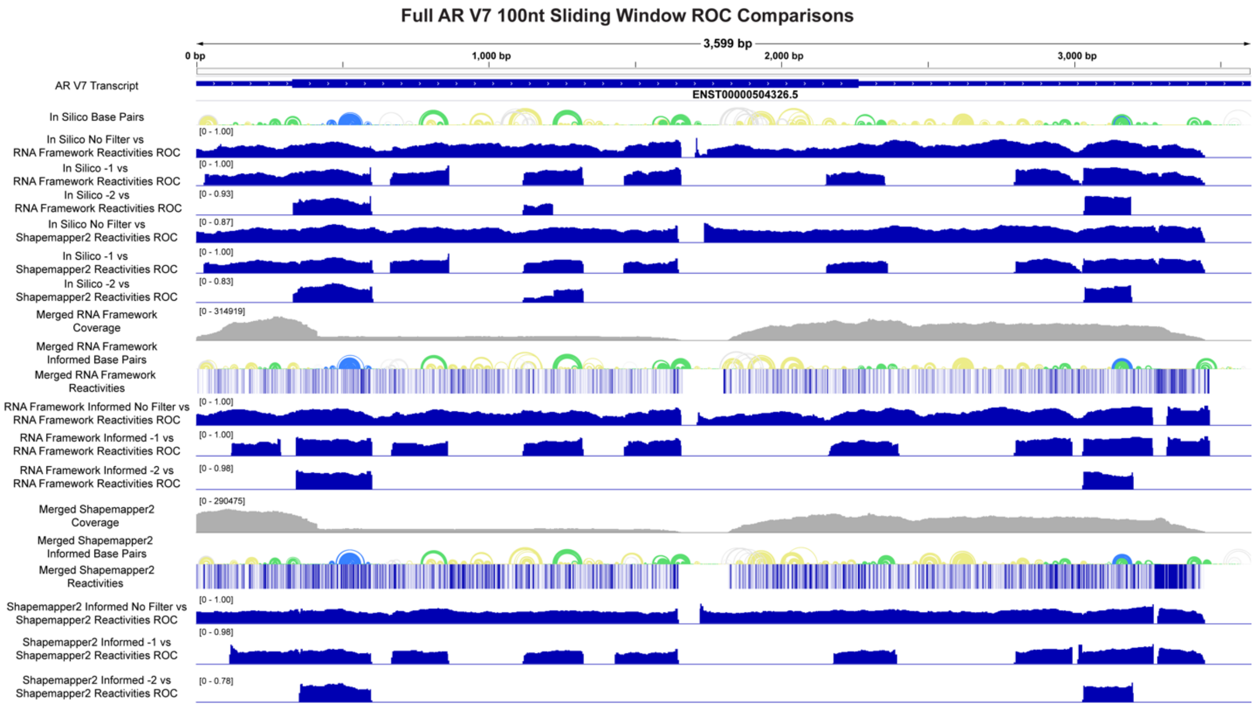

Figure S10. AR-V7 100nt sliding window ROC comparisons. All data from merged and individual sequencing analyses are overlayed against in silico ScanFold data, and 100nt sliding window ROC data. Data are presented for the AR-V7 transcript showing data for in silico ScanFold base pairs; in silico ScanFold no filter, -1, -2 z-score structures vs RNA Framework reactivities ROC data; in silico ScanFold no filter, -1, -2 z-score structures vs Shapemapper2 reactivities; merged RNA Framework coverage; merged RNA Framework informed ScanFold base pairs; merged RNA Framework reactivities; merged RNA Framework informed ScanFold no filter, -1, -2 z-score structures vs RNA Framework reactivities ROC data; merged Shapemapper2 coverage; merged Shapemapper2 informed ScanFold base pairs; merged Shapemapper2 reactivities; and merged Shapemapper2 informed ScanFold no filter, -1, -2 z-score structures vs Shapemapper2 reactivities ROC data. This data shows regions of high and low AUC values across the transcript. High values generally correspond to low z-score structures and low values generally correspond to high z-score structure. Similar to AR-FL this discrepancy among lower than expected AUC values in some low z-score areas agree with the DRACO results indicating structural heterogeneity and potential structural dynamics in these regions.

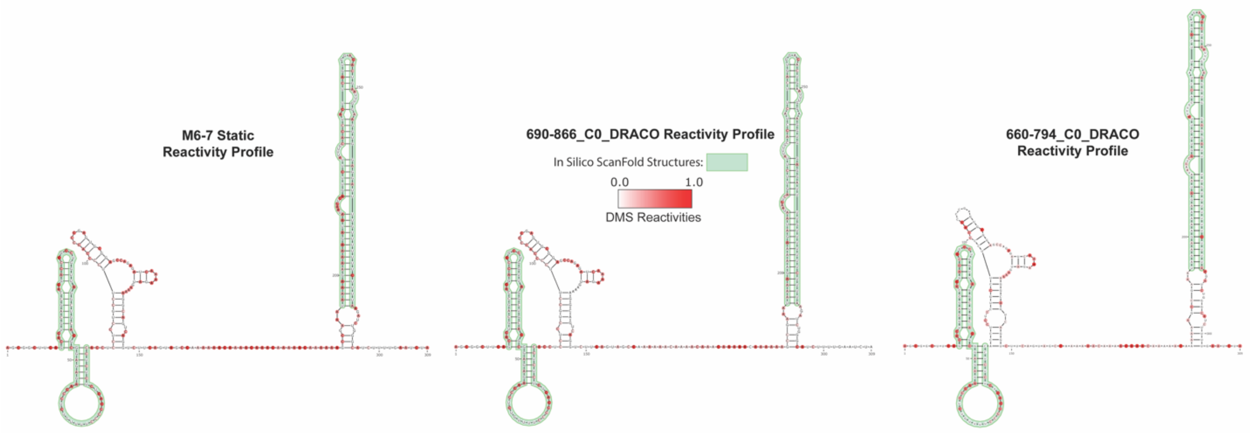

Figure S11. DRACO structural dynamics of the 5’UTR structure 6-7 region

DRACO was used in combination with DMS reactivities to identify dynamic regions throughout the AR-FL transcript. Looking at reactivity profiles encompassing predicted structures 6-7 in the 5'UTR, many unique profiles were extracted and incorporated into the surrounding static profile. Using a 120 nt pairing constraint, nearly identical structures were generated for each profile, and the ScanFold predicted hairpin structures 6-7 (green highlight) were maintained. The structure on the left was predicted by RNA Framework informed ScanFold using the average static reactivity profile. The middle and right structures were generated using two different DRACO reactivity profiles. The profiles used in these models as well as all other profiles, formed the same structures and maintained the ScanFold predicted hairpins (green highlight).

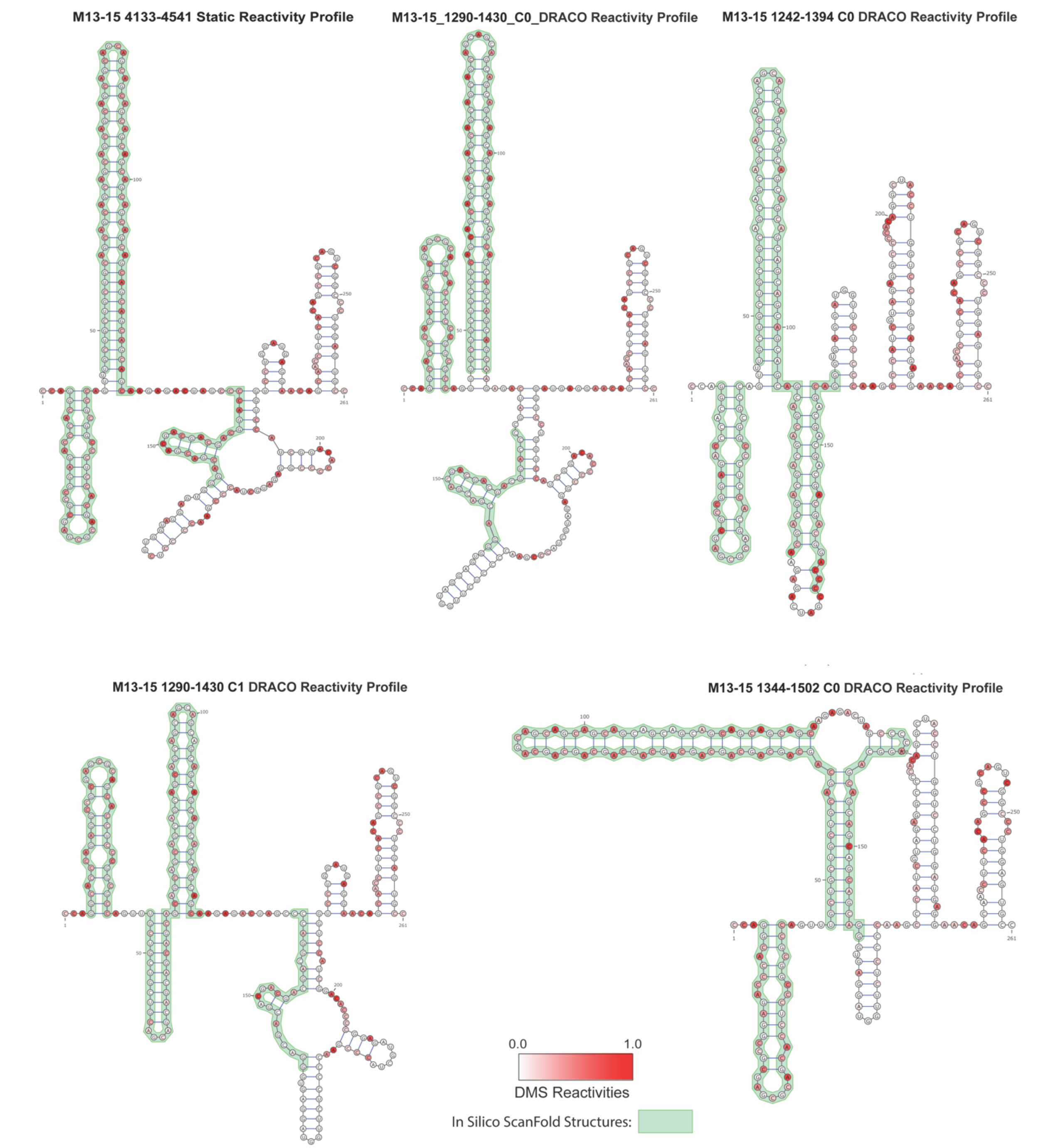

Figure S12. DRACO structural dynamics. DRACO was used in combination with DMS reactivities to identify dynamic regions throughout the AR-FL transcript. Looking at reactivity profiles encompassing predicted structures 13-15 in the CDS, a small number of unique profiles were extracted and incorporated into the surrounding static profile. Using a 120 nt pairing constraint, similar structures were generated for each profile, and the ScanFold predicted structures 13-15 (top left highlight) were maintained fully or partially. Here, the predominant changes were seen in the -2 z-score hairpin, however, this is not surprising due to the high ensemble diversity of the predicted structure and the nature of the CAG repeats that allow for base pairs to shift within this structure.

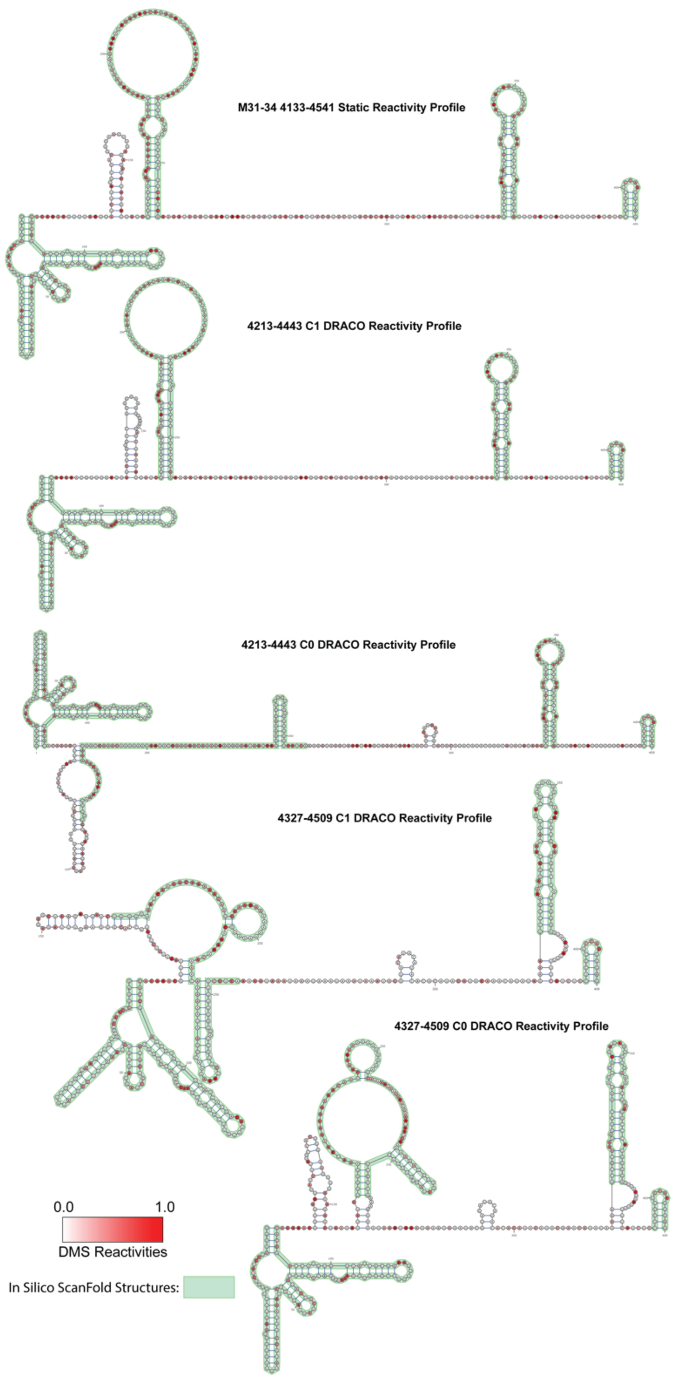

Figure S13. DRACO structural dynamics. DRACO was used in combination with DMS reactivities to identify dynamic regions throughout the AR-FL transcript. Looking at reactivity profiles encompassing predicted structures 31-34 in the 3'UTR, a small number of unique profiles were extracted and incorporated into the surrounding static profile. Using a 120 nt pairing constraint, similar structures were generated for each profile, and the ScanFold predicted structures 31-33 (top highlighted structures) were maintained fully or partially. Here, the predominant changes were seen in structure 32, where a fluctuation between a single hairpin or multi branch loop was seen. The sequence composition of this structure is predominantly A and U nucleotides and a large terminal loop with higher z-scores is predicted to form, which may explain this observed conformational switch.

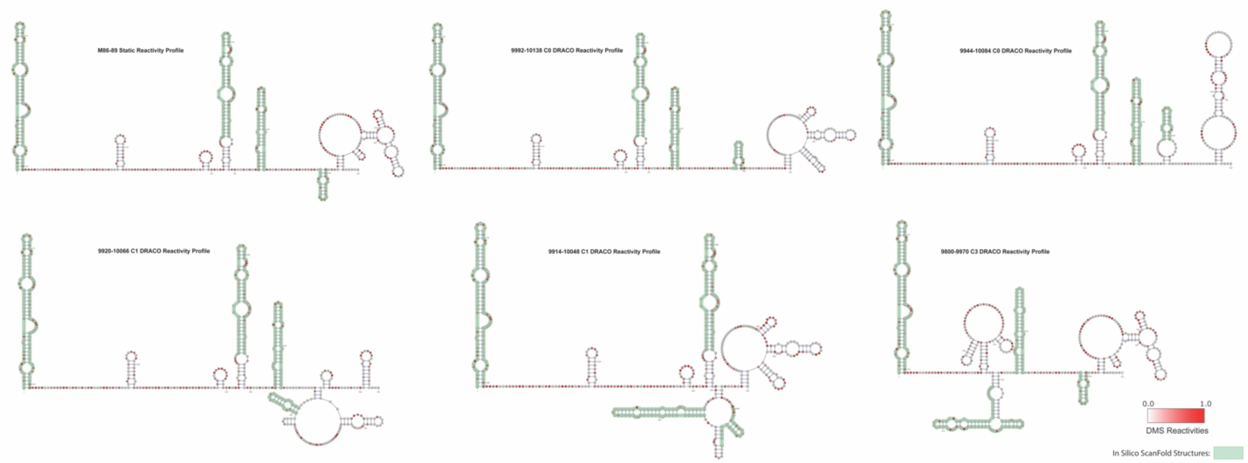

Figure S14. DRACO structural dynamics. DRACO was used in combination with DMS reactivities to identify dynamic regions throughout the AR-FL transcript. Looking at reactivity profiles encompassing predicted structures 86-89 in the 3'UTR, a small number of unique profiles were extracted and incorporated into the surrounding static profile. Using a 120 nt pairing constraint, similar structures were generated for each profile, and the ScanFold predicted structures 86-89 (top left highlighted structures) were maintained fully or partially. Here, the predominant changes were seen at the 3' end, outside of the ScanFold predicted structures. Preservation of these low z-score structures across multiple reactivity profiles offers further evidence that these structures may be functionally significant.

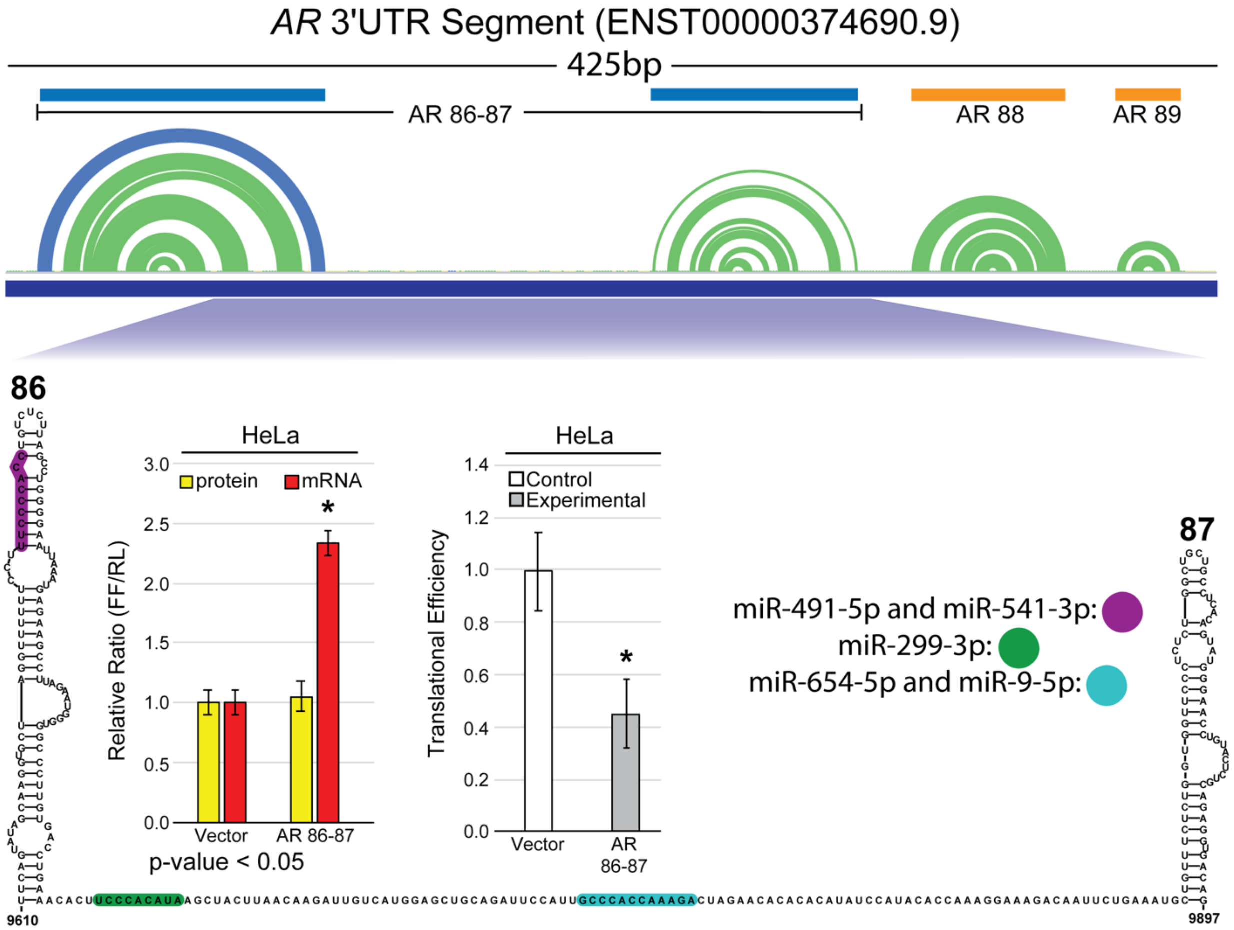

Figure S15. All structure function data for AR-FL 3'UTR structures 86-87. A 425 nt fragment of the AR-FL 3'UTR is shown. ScanFold predicted structures are represented as an arc diagram above the gene cartoon. Low z-score structures (blue and green arcs) are annotated with their number and a blue or orange box. Structures annotated with blue boxes are expanded and represented below the arcs as 2D models. For structures 86-87, the individual hairpins are modeled, numbered, and annotated with all relevant data. Structure 86 contains miR-491-5p and 541-3p sites (purple), and the single stranded region between structure 86 and 87 contains a miR-299-3p site (green), miR-654-5p site (blue), and a miR-9-5p site (blue). These structures were tested for function via dual luciferase assays and qPCR in HeLa cells. The changes in protein (yellow) and mRNA (red) levels compared to vector control can be seen in the left bar graph. Using the protein and mRNA levels, translational efficiency was calculated and plotted in the right bar graph. Asterisks represent a p-value < 0.05.
